# eEF2 improves dense connective tissue repair and healing outcome by regulating cellular death, autophagy, apoptosis, proliferation and migration

**DOI:** 10.1007/s00018-023-04776-x

**Published:** 2023-04-21

**Authors:** Junyu Chen, Jin Wang, Xinjie Wu, Nils Simon, Camilla I. Svensson, Juan Yuan, David A. Hart, Aisha S. Ahmed, Paul W. Ackermann

**Affiliations:** 1 Department of Molecular Medicine and Surgery, Center for Molecular Medicine, Karolinska Institutet, 171 76 Stockholm, Sweden; 2 Department of Pharmacology, School of Basic Medical Sciences, Xi’an Jiaotong University Health Science Center, Xi’an, Shaanxi 710061 People’s Republic of China; 3 The Key Laboratory of Tumor Molecular Diagnosis and Individualized Medicine of Zhejiang Province, Zhejiang Provincial People’s Hospital, Affiliated People’s Hospital, Hangzhou Medical College, Hangzhou, 310014 China; 4 Peking University China-Japan Friendship School of Clinical Medicine, Beijing, 100029 China; 5 Department of Physiology and Pharmacology, Center for Molecular Medicine, Karolinska Institutet, 171 76 Stockholm, Sweden; 6 Department of Cell and Molecular Biology, Karolinska Institutet, 17176 Stockholm, Sweden; 7 Department of Surgery, Faculty of Kinesiology, McCaig Institute for Bone and Joint Health, University of Calgary, Calgary, AB Canada; 8 Department of Physiology, University of Helsinki, Helsinki, Finland

**Keywords:** Mass spectrometry, Dense connective tissue repair, Fibroblast, eEF2, Cell biology, Regeneration

## Abstract

Outcomes following human dense connective tissue (DCT) repair are often variable and suboptimal, resulting in compromised function and development of chronic painful degenerative diseases. Moreover, biomarkers and mechanisms that guide good clinical outcomes after DCT injuries are mostly unknown. Here, we characterize the proteomic landscape of DCT repair following human Achilles tendon rupture and its association with long-term patient-reported outcomes. Moreover, the potential regulatory mechanisms of relevant biomarkers were assessed partly by gene silencing experiments. A mass-spectrometry based proteomic approach quantified a large number (769) of proteins, including 51 differentially expressed proteins among 20 good versus 20 poor outcome patients. A novel biomarker, elongation factor-2 (eEF2) was identified as being strongly prognostic of the 1-year clinical outcome. Further bioinformatic and experimental investigation revealed that eEF2 positively regulated autophagy, cell proliferation and migration, as well as reduced cell death and apoptosis, leading to improved DCT repair and outcomes. Findings of eEF2 as novel prognostic biomarker could pave the way for new targeted treatments to improve healing outcomes after DCT injuries.

**Trial registration**: NCT02318472 registered 17 December 2014 and NCT01317160 registered 17 March 2011, with URL http://clinicaltrials.gov/ct2/show/NCT02318472 and http://clinicaltrials.gov/ct2/show/study/NCT01317160.

**Supplementary Information:**

The online version contains supplementary material available at 10.1007/s00018-023-04776-x.

## Introduction

Human dense connective tissues (DCTs) are matrix-rich tissues which provide vital support and protection to other tissues and organs in the body. However, the reparative process after an injury, especially after injury to DCTs such as tendon, ligament, meniscus and intervertebral disc, can lead to considerable individual variation in long-term patient outcomes. Such variations often result in compromised function, chronic pain and degenerative musculoskeletal diseases for many patients. Knowledge of the regulatory mechanisms responsible for such variability in regenerative outcomes, as well as identification of validated biomarkers that can be used as predictors of DCT healing are still mostly lacking [1–4].

Prior studies in animals have utilized proteomics to delineate the healing of DCT [5–8]. However, there is a very limited number of reports examining how the human proteome changes in this process. One human study has compared the proteomes of keloid scars and healthy skin [9]. The low number of human proteomic studies of DCT healing presumably relate to problems in obtaining large numbers of clinical specimens and subsequently integrating the findings with patient-reported outcomes. However, some aspects of these limitations can potentially be overcome using recent technological developments in the field of mass spectrometry (MS)-based proteomics which have considerably improved the sensitivity, completeness and quantitative accuracy of this technique for investigating human diseases [10, 11]. Our recent findings using MS to analyze the proteomic landscape of human tendon repair in patients with Achilles tendon rupture (ATR) with high quality and sensitivity [12] demonstrate the utility of such approaches.

The Achilles tendon is the largest and thickest dense connective tissue in the human body, as well as one of the most commonly injured DCT [13]. Patients with ATR also exhibit considerable variability in patient-reported outcomes after injury [14]. Previous studies indicated that improved patient-reported outcomes after ATR and other DCT injuries are associated with higher production of collagen type I (Col1) [1, 15, 16]. Col1, produced by fibroblast cells [17], is the most abundant protein in these tissues, bringing structure and strength to the matrix of DCTs. Thus, specific biomarkers with strong association to Col1 synthesis would provide a tool to monitor improvement in DCT repair. However, the regulatory mechanisms regarding Col1 production in fibroblast cells during DCT repair remain far from being fully understood [18].

To test the hypothesis that a proteomic approach would lead to the identification of novel prognostic biomarkers of early healing efficacy, we aimed to characterize the proteomic components of human DCT repair by combining MS-based proteomics with validated patient-reported outcomes. The present studies also aimed to assess the best prognostic biomarker utilizing tissue biopsies obtained from ATR patients at the time of repair surgery, and with good and poor 1-year clinical outcomes. Further, the regulatory mechanisms involved for the identified biomarkers on Col1 synthesis were explored in primary fibroblast cells and in fibroblast cell lines by gene silencing to knock down the expression of specific molecules. The findings indicate that eukaryotic elongation factor-2 (eEF2) is such a biomarker for prognosis of good outcomes following injury to a DCT.

## Materials and methods

### Ethical approval and consent to participate

This study was conducted with the approval from the Regional Ethical Review Committee in Sweden and followed all guidelines according to the Declaration of Helsinki. Written consent was obtained from all patients.

### Patient eligibility criteria and randomization

This was a retrospective study of 40 patients suffering from an acute Achilles tendon rupture (ATR) who underwent reconstruction surgery at the Karolinska University Hospital. The majority of the ATR-cases (90%) were sports-related. During the surgery, tendon biopsies were taken from the ruptured area and stored at minus 80 °C for future analysis. Patients were randomly selected from a patient cohort who participated in randomized control trials at the hospital. All the participants received the same anesthetic and surgical procedure to repair mid-substance tears within 2–7 days of injury and followed exclusion criteria as reported previously [3, 16].

### Patient-reported and functional outcome

The patient-reported outcomes were evaluated 1-year postoperatively using validated questionnaires: Achilles Tendon Total Rupture Score (ATRS), Foot and Ankle Outcome Score (FAOS) and functional outcome was measured using the Heel-rise Test (HRT). All the methodology, rules and regulations of patient outcome measurement followed the same description as in our previous studies [1, 3, 16, 19].

### Mass spectrometry (MS)

#### Protein extraction and digestion of tissue samples

Pulverized patient tissue samples were solubilized in 8 M urea and 100 mM NaCl with 1% ProteaseMAX (Promega) in 100 mM ammonium bicarbonate (AmBic) and mixed vigorously. Low binding silica beads (400 µm, Ops Diagnostics, Lebanon NJ) were added to each sample and vortexed at high speed. Subsequently, samples were quickly frozen and subsequently then thawed and subjected twice to disruption of the tissue on a Vortex Genie disruptor for 2 min before addition of AmBic, urea and NaCl. Following centrifugation, the supernatant was transferred to new tubes and 50 mM AmBic was added and the mixture was vortexed vigorously.

Proteins were reduced with 100 mM dithiothreitol in 50 mM AmBic, incubated at 37 °C and alkylated with 100 mM iodoacetamide in 50 mM AmBic in the dark. Proteolytic digestion was performed overnight. The reaction was stopped with concentrated formic acid and the samples were then cleaned on a C18 Hypersep plate (bed volume of 40 µL, Thermo Scientific) and dried in a vacuum concentrator (miVac, Thermo Scientific).

### RPLC-MS/MS analysis

Briefly, reversed phase liquid chromatographic separations of peptides were performed on a C18 EASY-spray and C18 trap columns connected to an Ultimate 3000 UPLC system (Thermo Scientific). Mass spectra were acquired on an Q Exactive HF mass spectrometer (Thermo Scientific), targeting 5 × 10^6^ ions with maximum injection time of 100 ms, followed by data-dependent higher energy collisional dissociation (HCD) fragmentations from precursor ions with a charge state.

### Proteomic data analysis, protein identification and quantification

Raw files were imported to Proteome Discoverer v2.3 (Thermo Scientific) and analyzed using the SwissProt protein database with the Mascot v 2.5.1 (MatrixScience Ltd., UK) search engine. MS/MS spectra were matched with The Human Uniport database (last modified: 3 September 2020; ID: UP000005640; 75,777 proteins) using the MSFragger database engine [20].

Protein abundance was calculated based on normalized spectrum intensity (LFQ intensity), and an intensity-based absolute quantification (iBAQ) algorithm was used for normalization[21]. A *p* value < 0.05 and fold change (FC) > 2 were set as significant threshold to identify differentially expressed proteins between good and poor outcome patients. Gene Ontology (GO) annotation and KEGG database were used to identify the biological functions of enriched proteins. Enrichment analysis was performed with GO annotations on STRING v11.0 (http://string-db.org) database for the confirmation of protein–protein interaction network among subgroups. Gene set enrichment analysis (GSEA) and single-sample GSEA (ssGSEA), based on the GO and KEGG databases were used to detect potential pathways involved in the candidate identified biomarkers.

### Cell culture

The use of fibroblast cells for connective tissue studies has been previously reported [22]. Human primary fibroblasts (#C-12302, Promo Cells) and the fHDF/TERT166 human foreskin fibroblast cell line (#CHT-031–0166, Evercyte, Austria) were used for the in vitro studies and for confirmation of the in vivo findings. Cells were cultured in Dulbecco’s Modified Eagle Medium/F12 (DMEM/F12, Gibco) supplemented with 10% fetal bovine serum (FBS, Gibco) and 1% penicillin–streptomycin (PEST) (Gibco) at 37 °C in a humidified atmosphere containing 5% CO_2_. After reaching 80–90% confluency, cells were dissociated with 0.25% trypsin- ethylenediaminetetraacetic acid (Trypsin–EDTA) (Thermo Fisher Scientific) and seeded in 12-well plates at 2.5 × 10^5^ cells/well supplemented with DMEM/F-12 medium for further experiments.

### Cell transfection

Human fibroblasts were seeded and transfected with eEF2 silencing RNA (si-eEF2) (#4392420, Ambion) to detect the potential role of eEF2 in tendon healing. Cells were stimulated with 10 ng/mL TNF for 24 h before being transfected with si-eEF2. Cells exposed to silencing RNA for eEF2 and negative controls were transfected for 48 h at a final concentration of 100 nM in each well using Lipofectamine™ 3000 transfection reagent (Thermo Fisher Scientific) and diluted with Opti-MEM reagent.

### Inflammatory fibroblast injury model

Recombinant human tumor necrosis factor alpha (TNF-α) (#300-01A, PeproTech) was used to establish an inflammatory insult to the fibroblasts after transfection. Bovine serum albumin (BSA), 0.2% was used for dissolving the TNF, and 10 ng/mL was used for fibroblast stimulation [23].

### Immunohistochemistry (IHC) and immunofluorescence (IF)

Human tendon biopsies were cut into 7 μm sections using a cryostat. Briefly, tissue sections were incubated with 2% H_2_O_2_ and blocked with 5% bovine serum albumin (BSA) at room temperature. Monoclonal antibody to eEF2 (#MA5-42937; Thermo Fisher Scientific) and a secondary antibody, goat anti-rabbit (#31460; Thermo Fisher Scientific, 1:250) was used for immunolocalization. The bound antibody was visualized with diaminobenzidine (DAB, Vector Laboratories, Inc. Burlingame, CA, USA) and counterstained with Hematoxylin (Vector Laboratories, Inc. Burlingame, CA, USA). This step was followed by dehydration in alcohol and xylene before mounting with pertex (Sigma). To demonstrate specificity of staining, primary antiserum was either omitted or replaced by normal rabbit IgG.

For fluorescence staining, slides were blocked with 2% BSA in 1 mol/L phosphate buffered saline (PBS) and subsequently incubated with the primary antibody against eEF2 overnight at 4 °C and then incubated with Alexa Fluor 488-labeled Goat anti-rabbit IgG secondary antibody at room temperature. All the slides were stained with antifade reagent (with DAPI) and mounted. Negative controls were prepared by omitting the primary antibody or including a rabbit IgG1 (M5284, 1:100 dilution) isotype control. Data analysis was performed using ImageJ software.

Cells were plated on the glass chamber slides and treated with starved stimulation for autophagy, eEF2 siRNA and 3-Methyladenie (3-MA), respectively. Next, the cells were washed, fixed with 4% paraformaldehyde (PFA), blocked with 5% BSA in PBS, permeabilized with 0.1% Triton X-100, incubated with anti-Col1a1 antibody (CST) and followed by Alexa-Fluor-488 conjugated secondary antibody (#A-11008; Thermo Fisher Scientific). All the slides were stained with the antifade reagent (with DAPI) and mounted. The single-stained and merged images were performed using a Zeiss LSM 880 confocal microscopy (without AiryScan, CMM Karolinska Institutet), and the data was analyzed using ImageJ software.

### Cell proliferation assay

A Click-iT^®^ EdU AlexaFluor 488^®^ Imaging Kit (Thermo Fisher Scientific) was used to identify the changes in proliferation rates of human fibroblast primary cells and the cell line. Normal, transfected and 3-MA stimulated cells were incubated with EdU media for 2 h before fixation with 4% PFA. Each section was incubated with freshly prepared Click-iT^®^ reaction cocktail as instructed in the kit. BSA (3%) in PBS was used to wash the slides twice for 5 min. Cell nuclei were stained with Hoechst 33342 for 5 min and the slides were subsequently washed with PBS for 2 × 5 min. All the steps of the EdU assay were performed while avoiding light and the slides were never allowed to dry out.

### Cell apoptosis assay

Live/Dead™ Cell Imaging Kit and CellEvent™ Caspase-3/7 Green Detection Reagent (Thermo Fisher Scientific) were used for detection of cell apoptosis based on the manufacturer instructions. Cells were treated with si-eEF2, fixed with 4% PFA (specifically for casepase-3/7) and stained using an annexin V-FITC, PI and Caspase-3/7 green detection reagent in dark. Hoechst 33342 was used for cell nucleus staining. Finally, cells were assessed and quantified using the fluorescence microscope. Image J and SoftMax Pro 7.0.3 were used to perform the data analysis.

### Scratch wound assay

A cell scratch assay was used to explore the role of eEF-2 on cell migration. Based on an unchallenged fibroblast model, cells were seeded in six-well plates and a scratch was created using a pipette tip on the confluent monolayer cell culture. Next, cells were transfected with si-eEF2 and compared with a control group, all the subgroups were treated with no-FBS to block cell proliferation. After 24 h, light microscopy and ImageJ were used to assess the results.

### Western blot analysis

Briefly, 5 μg of protein per well was loaded, separated by gel electrophoresis (4–12% Bis–Tris, Invitrogen), and transferred to nitrocellulose membranes. The membranes were incubated with 5% nonfat milk in 1 × tris-buffered saline with 0.1% tween (TBST) to block unspecific binding sites, and the membranes were subsequently covered with different primary antibodies overnight at 4 °C (eEF2 0.1 μg/mL, Col1a1 0.1 μg/mL, β-actin 0.02 μg/mL, GAPDH 0.01 μg/mL, Cell Signaling). After washing the membranes, they were incubated with secondary antibody conjugated to HRP (anti-rabbit IgG and anti-mouse IgG; Cell Signaling). The chemiluminescence signal was initiated using SuperSignal West Pico PLUS kit (Thermo Fisher Scientific). Chemiluminescence was identified and quantified using biorad ChemiDoc MP Imaging and analysis was performed in Image Lab. Immunopositive bands were normalized with the bands of GAPDH or β-actin and final results were presented as fold change from control values.

### Statistical analysis

Statistical analysis and data plotting were performed with SPSS (IBM SPSS, v26.0), GraphPad Prism 8.0 and R. Skewness was checked for all the variables. Standard descriptive statistics were used to summarize clinical variables such as mean and SD. Differential comparison of protein expressions was calculated using the unpaired Mann–Whitney *U* test, a *p* value < 0.05 and FC > 2 were considered as statistically significant. The patient outcome measurements were associated with the corresponding proteomic files by means of univariate analysis. Outcome measures that were significant in univariate analysis were subsequently used as dependent variables in a multiple-linear regression model with independent variables, and an adjusted *p* value < 0.05 was set as the significance threshold.

## Results

### Patient cohort

To create a tissue atlas of good and poor connective tissue repair, surgical biopsies from a total of 40 patients with ATR were collected, of which 20 patients were assessed to have had a good clinical outcome and the other 20 patients were evaluated as to having a poor clinical outcome according to the 1-year validated patient-reported outcomes [19]. Patient characteristics and 1-year patient-reported and functional outcomes are presented in Table 1.

**Table 1:** Patient characteristics and outcome data

Variables	Group	Good outcome	Poor outcome	*p* Value
*N* (M: F)				
32:8	40	20	20	
Age (years)	40 (26, 65)	40 (26, 65)	39 (28, 59)	ns
BMI (kg/m^2^)	25.4 (19.6, 34.7)	24.6 (19.6, 34.7)	25.4 (20.6, 30.6)	ns
TTS (h)	85.8 (24.5, 158.7)	87.5 (24.5, 158.7)	85.3 (41.0, 135.6)	ns
ATRS	76 (40, 100)	95 (87, 100)	63 (40, 78)	< 0.001
Limitation in calf strength	8 (1, 10)	9 (7, 10)	5 (1, 8)	< 0.001
Tiredness in the calf	8 (3, 10)	10 (7, 10)	5 (3, 8)	< 0.001
Stiffness in the calf	8 (2, 10)	10 (5, 10)	5 (2, 8)	< 0.001
Pain in the calf	10 (3, 10)	10 (9, 10)	9 (3, 10)	< 0.001
Activity of daily life	9 (4, 10)	10 (9, 10)	8 (4, 10)	< 0.001
Walking on uneven surface	10 (3, 10)	10 (7, 10)	8 (3, 10)	< 0.001
Limitation on walking in stairs	9 (4, 10)	10 (9, 10)	8 (4, 10)	< 0.001
Limitation on running	8 (0, 10)	10 (8, 10)	6 (0, 9)	< 0.001
Limitation on jumping	7 (2, 10)	10 (7, 10)	4 (2, 9)	< 0.001
Loss in physical work	9 (3, 10)	10 (9, 10)	8 (3, 10)	< 0.001
FAOS				
Pain	100 (69, 100)	100 (89, 100)	93 (69, 100)	0.007
Symptom	93 (39, 100)	100 (75, 100)	89 (39, 100)	0.009
Activity of daily life	100 (88, 100)	100 (91, 100)	98 (88, 100)	0.012
Sport and recreation	88 (45, 100)	100 (75, 100)	75 (45, 90)	< 0.001
Foot-and ankle-related QOL	69 (31, 100)	91 (69, 100)	56 (31, 88)	< 0.001
HRT (LSI%)				
Concentric power	80.8 (23.5, 189.9)	80.6 (52.9, 115.9)	82.1 (23.5, 189.9)	ns
Total work	71.5 (24.3, 288.0)	77.7 (57.5, 119.6)	68.2 (24.3, 288.0)	ns
Repetition	90.5 (45.8, 275)	96.7 (71.4, 114.3)	82.7 (45.8, 275.0)	ns
Average height	81.8 (37.6, 110.5)	81.8 (65.1, 110.5)	81.9 (37.6, 104.0)	ns

The clinical and functional characteristics of ATR patients included in study

*TTS* time to surgery; *BMI* body mass index; *ATRS* Achilles tendon Total Rupture Score (0–100, and 0–10 for each subscale, worst = 0); *FAOS*  foot and ankle outcome score (0–100, worst = 0 for each category); *HRT* heal rise test (0–100, worst = 0 for each category); *QOL* quality of life; *LSI* limb symmetry index. Data presented as median with lower and upper interquartile ranges. Comparison between good versus poor outcome was measured by Mann–Whitney *U* test

A *p* value < 0.05 was set for statistical significance between group

### Quantitative proteomic characterization of good and poor outcome patients

**Fig. 1:**
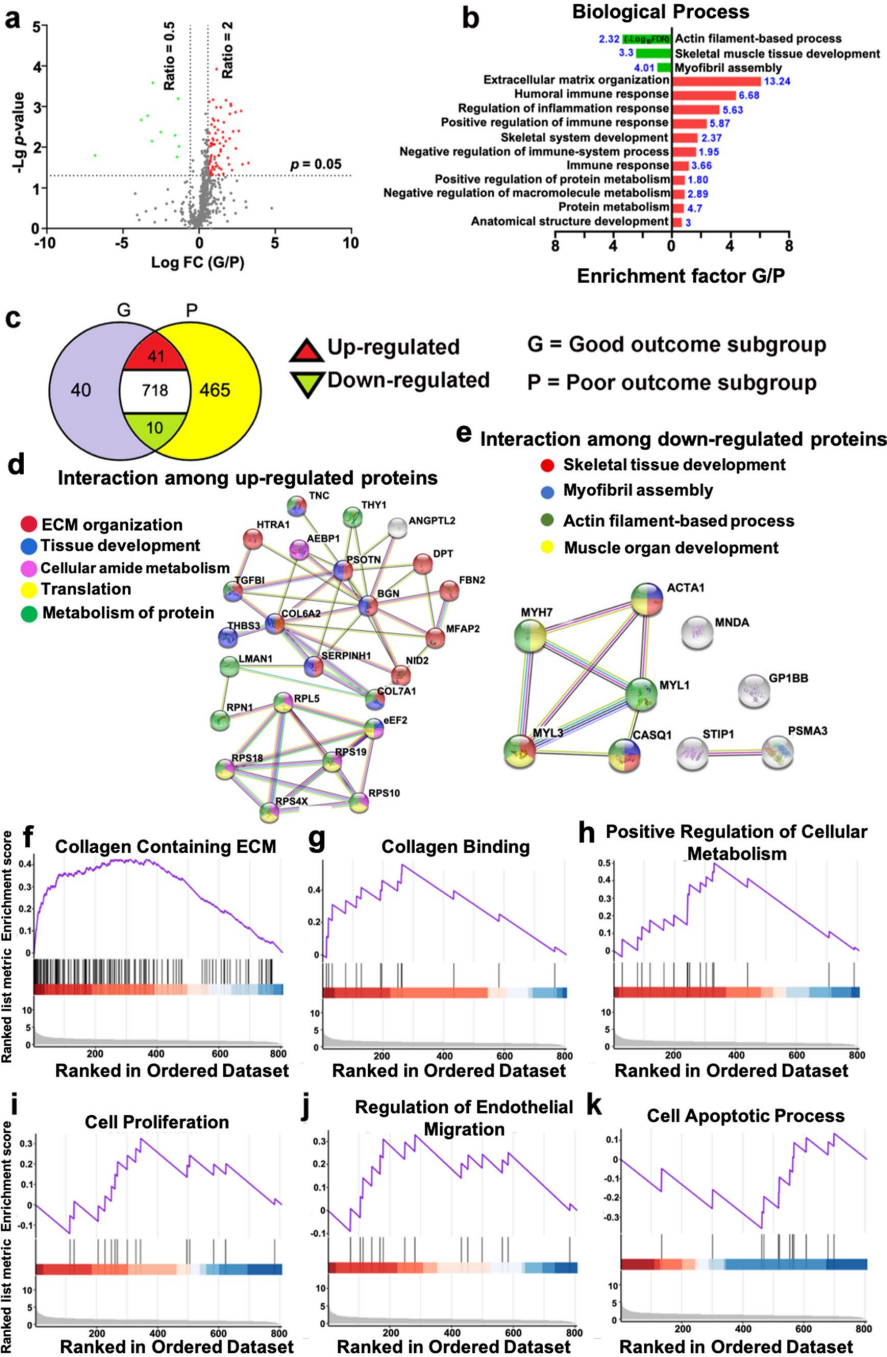
Quantitative proteomic file of injured human Achilles tendon. **a** Volcano diagram with differentially expressed proteins. The *X* coordinate represents Log_2_ fold change (FC) and the *Y* coordinate to −Log_10_ (*p*-value). Each dot represents a protein with red = upregulated, green = downregulated and, black = non-differentially expressed proteins. **b** Bar plot of differentially expressed proteins with matched biological processes. The length of the bar represents the percentage of proteins which are enriched in each pathway; −log10 FDR was used as reliability, red = upregulated while green = downregulated proteins; The enrichment factor displays the ratio of the pathway related proteins in relation to the whole identified proteome. **c** Venn plot of overlapping and distinct proteomes of patients with good (G) and poor (P) outcome. *n* = 20 in each group; Protein–protein interactions among **d** upregulated and **e** downregulated proteins. STRING v11.0 was used to analyze functional enrichment. Relevant biological processes are presented with different colors; **f**–**k** GSEA plots of the highly involved pathways related to differentially expressed proteins

To identify proteins that are potentially prognostic of good and poor Achilles tendon repair, proteins from the tissue samples were extracted and subsequently separated using reverse phase liquid chromatography. The proteome of the extracted proteins was developed using mass spectroscopy as detailed in the “Materials and methods” section. The proteomic data were then grouped based on the 1-year clinical outcomes for the patients [19]. The computational data analysis detected 855 unique proteins, including 769 shared proteins across the good and poor outcome groups (Fig. 1a). Among the shared proteomic file, 51 differentially expressed proteins were identified with 10 down- and 41 upregulated proteins in good when compared with poor outcome subgroup (Fig. 1b). By analysis of enrichment factor, it was observed that the most enriched processes for the downregulated proteins included myofibril assembly and skeletal tissue development. The upregulated proteins were mostly involved in extracellular matrix (ECM) organization, collagen metabolism, and inflammatory and immune responses (Fig. 1b).

A highly enriched protein–protein interaction among the 10 downregulated proteins was detected, highlighting potential biological processes of skeletal tissue development, myofibril assembly and muscular development (Fig. 1e). The protein–protein interaction among 41 upregulated markers was also identified, which revealed that these proteins are mainly involved in ECM organization, tissue development and protein metabolism (Fig. 1d). GSEA of the 51 (10 down- and 41 upregulated proteins) differentially expressed proteins detected collagen binding, ECM regulation, as well processes involved in cell migration and proliferation, and apoptotic processes as potential enriched pathways (Fig. 1f–k). All the potential pathways based on GSEA analysis were collected and are shown in the supplementary material (Supplementary Fig. 1).

### Detection of predictive biomarkers for dense connective tissue repair

**Fig. 2:**
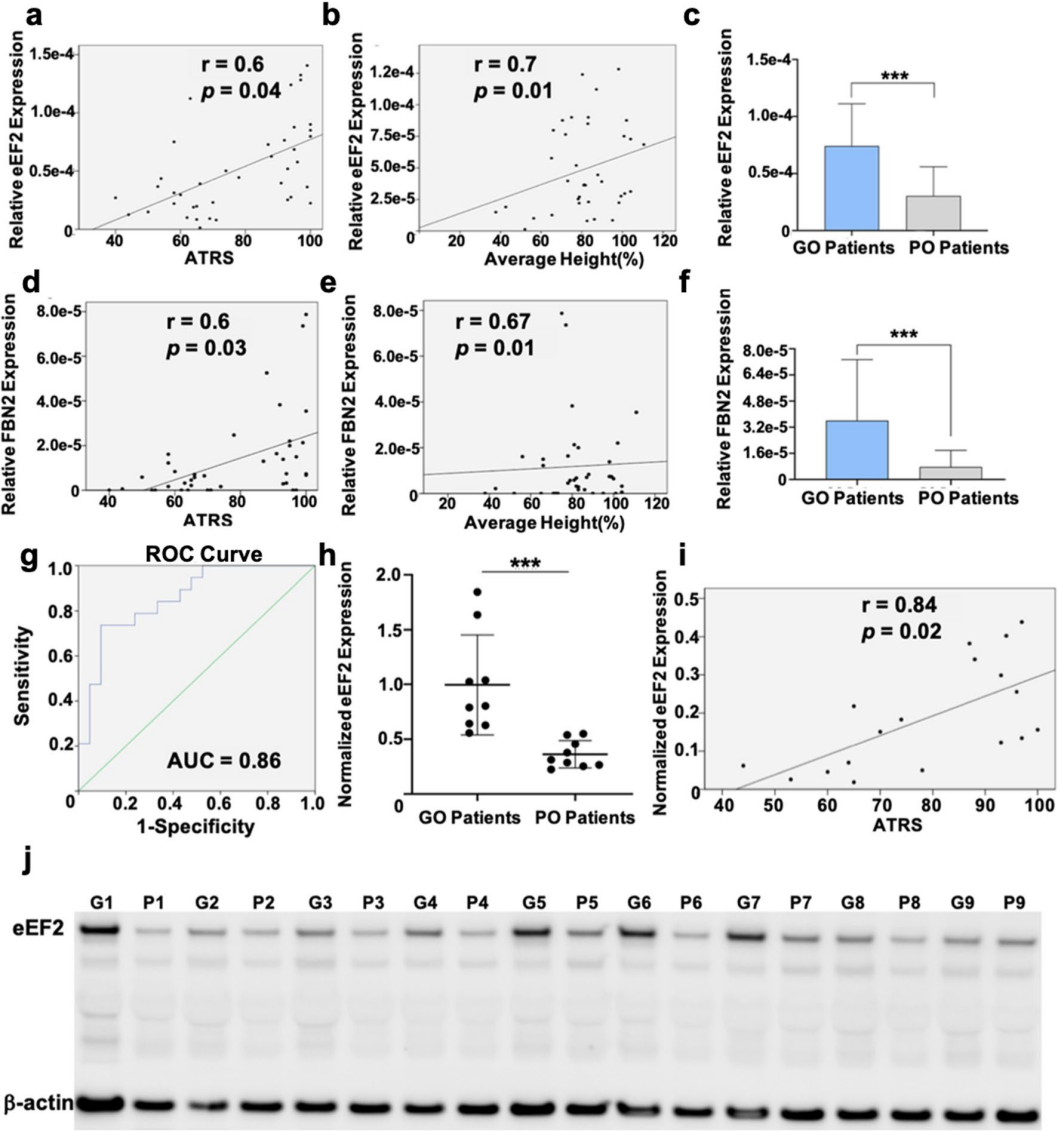
Prognostic biomarker selection and verification in injured Achilles tendon tissues. Association of eEF2 expression with **a** ATRS and, **b** average heal rise, *n* = 40; **c** eEF2 expression between good outcome (GO, *n* = 20) and poor outcome (PO, *n* = 20) patients, data presented as mean $\pm$ SD, ***p < 0.001; Association among FBN2 and **d** ATRS and, **e** average heal rise, *n* = 40; **f** FBN2 levels among good outcome (GO, *n* = 20) and poor outcome (PO, *n* = 20) patients, data presented as mean $\pm$ SD, ***p < 0.001; **g** AUC for eEF2 to report its predictive significance, *n* = 40; **h** Semi-quantitative analysis of eEF2 western blot analysis among good outcome (GO, *n* = 9) and poor outcome (PO, *n* = 9) patient samples, data presented as mean $\pm$ SD, **p < 0.01, *n* = 9 per group; **i** Positive association between eEF2 and 1-year healing outcome based on western blot analysis, *n* = 18; **j** Western blot image of eEF2 and beta-actin (β-actin) in good (G) and poor (P) outcome patients

To identify potential prognostic biomarkers, a multiple-regression analysis using the proteomic and clinical and functional data were performed. As a result, two upregulated proteins, eEF2 (Fig. 2a–c) and fibrillin-2 (FBN2) (Fig. 2d–f) were identified, both of which were positively associated with improved ATRS and average heel rise height. However, only eEF2 with an area under the curve (AUC) value of 0.86 (> 0.85), as compared to FBN2 with an AUC of 0.69, exhibited a strong prediction of good clinical outcome (Fig. 2g).

To confirm the proteomic data findings for eEF2, western blot analysis was conducted on tissue biopsies collected from same patient cohort used for MS which revealed significantly higher eEF2 levels in good compared to poor outcome patients (Fig. 2k). We further studied the ratio of phosphorylated-eEF2 (p-eEF2)/total eEF2 as eEF-2 kinase can modulate the activity of p-eEF2 [24]. However, no statistical difference was detected among good and poor outcome patients based on both MS and WB data analysis (Supplementary Fig. 2). Furthermore, a strong positive association was observed between eEF2 and 1-year clinical outcome (Fig. 2i). Together with a strong prognostic significance of the AUC value of 0.9 (Supplementary Fig. 2), eEF2 was selected as the best prognostic biomarker of Achilles tendon repair and subjected to further mechanistic analyses.

### eEF2 regulates collagen expression during dense connective tissue repair

**Fig. 3:**
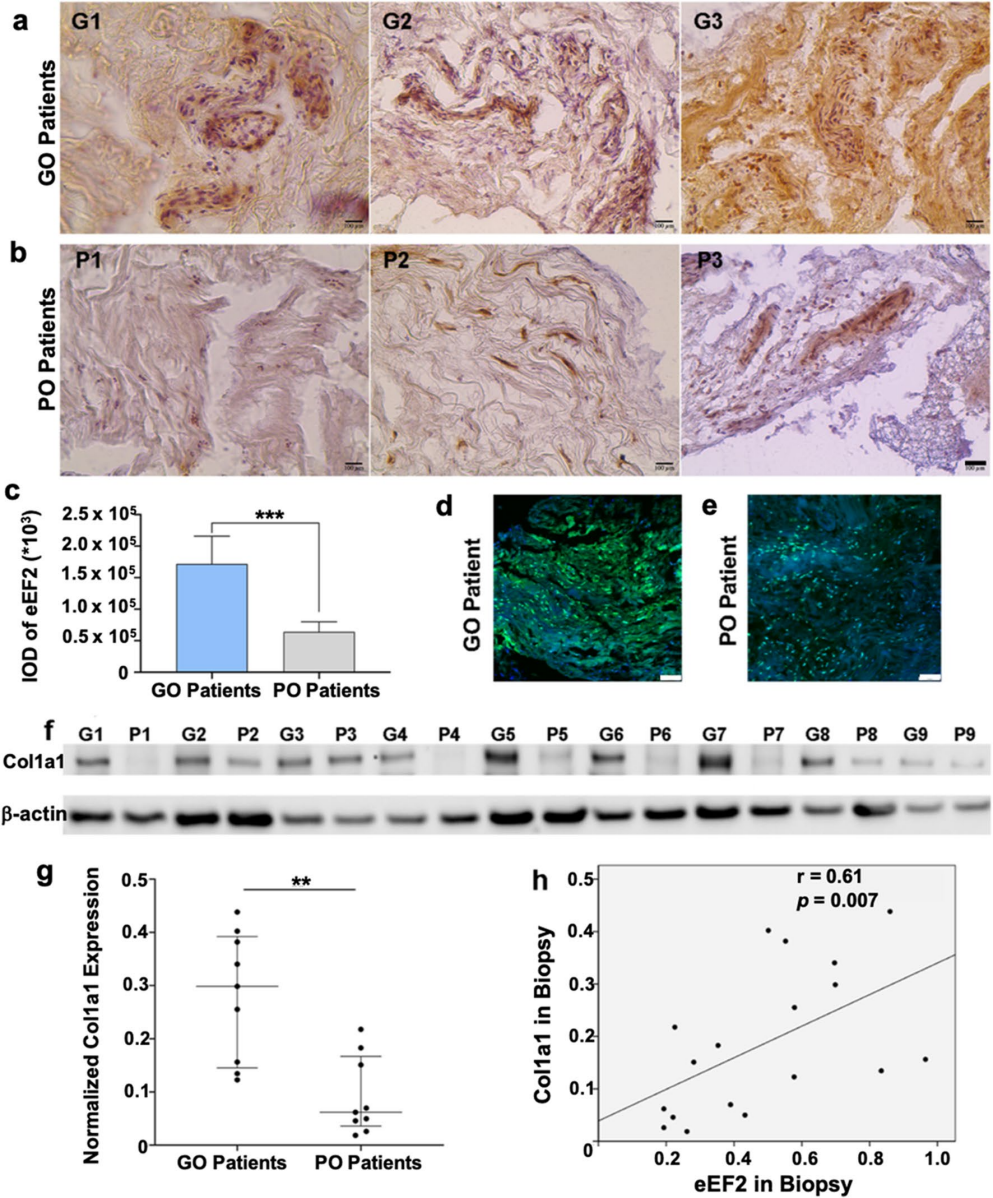
eEF2 expression and localization in tissue biopsies and association with Col1a1. Immunohistochemistry of the eEF2 in **a** good outcome (GO) and, **b** poor outcome (PO) patients, Scale bars = 100 µm; **c** semi-quantitative analysis of eEF2 expression in the good outcome (GO) and poor outcome (PO) patient samples presented as integrated optical density (IOD), data reported as mean $\pm$ SD, ***p < 0.001, *n* = 9 per group. eEF2 immunofluorescence signal in a **d** good outcome (GO) and, **e** poor outcome (PO) patient sample. Scale bars = 100 µm; **f** Western blot analysis and **g** semi-quantitative analysis of Col1a1 expression in good and poor outcome patients. Signal intensity was used for quantitative analysis, and the intensity of the house-keeping gene (beta-actin) used for normalization; **h** association among eEF2 and Col1a1 expression, *n* = 18

To visualize the localization and to confirm eEF2 expression in tissue biopsies, immunohistochemistry (IHC) and immunofluorescence (IF) analysis were performed. Both IHC (Fig. 3a–c) and IF (Fig. 3d, e) analyses clearly demonstrated higher eEF2 expression in the good compared to the poor outcome patients. Further, IHC and IF based semi-quantitative analyses demonstrated that eEF2, which is both a nuclear and cytoplasmic protein [25, 26], was mostly located in the ECM area of the tendon tissue (Fig. 3a, b, d, e). To identify how eEF2 improves healing in connective tissues after injury, the association between eEF2 and a classical marker of tendon repair, Collagen type I a1 (Col1a1) was analyzed. Western blotting from protein lysates generated from biopsies used for the MS analysis were used for Col1a1 expression in patients with good and poor outcome. The analysis demonstrated higher Col1a1 expression among patients with better outcome (Fig. 3f, g), in accordance with the findings for eEF2 (Fig. 2j). The upregulation of both eEF2 (Fig. 2h, j) and Col1a1 (Fig. 3f, g) was observed in the good compared with the poor outcome group. Interestingly, a strong relationship was observed between eEF2 and Col1a1 (*r* = 0.61, *p* = 0.007, Fig. 3h) levels, suggesting that eEF2 may mediate Col1a1 production to enhance tendon repair.

### eEF2 enhances dense connective tissue repair during inflammation by improving autophagy

Autophagy, which is a self-renewal mechanism that can degrade and recycle cellular components, plays an essential role in various phases of wound healing [27–29]. Specifically, in the inflammatory phase autophagy prevents excessive inflammation and can also regulate collagen synthesis [30]. The bioinformatic analysis, based on the proteomic data also identified that autophagy and collagen expression were improved by eEF2, highlighting a potential role of this novel biomarker during the inflammatory stage of tendon repair (Supplementary Fig. 3). Thus, an inflammatory fibroblast injury model was created to confirm and further explore whether eEF2 regulates collagen production by mediating autophagy during the inflammatory phase of repair. An inflammation-induced decline of Col1a1 was detected, and Col1a1 expression was reduced following silencing of eEF2 expression in the cells (Supplementary Fig. 4).

**Fig. 4:**
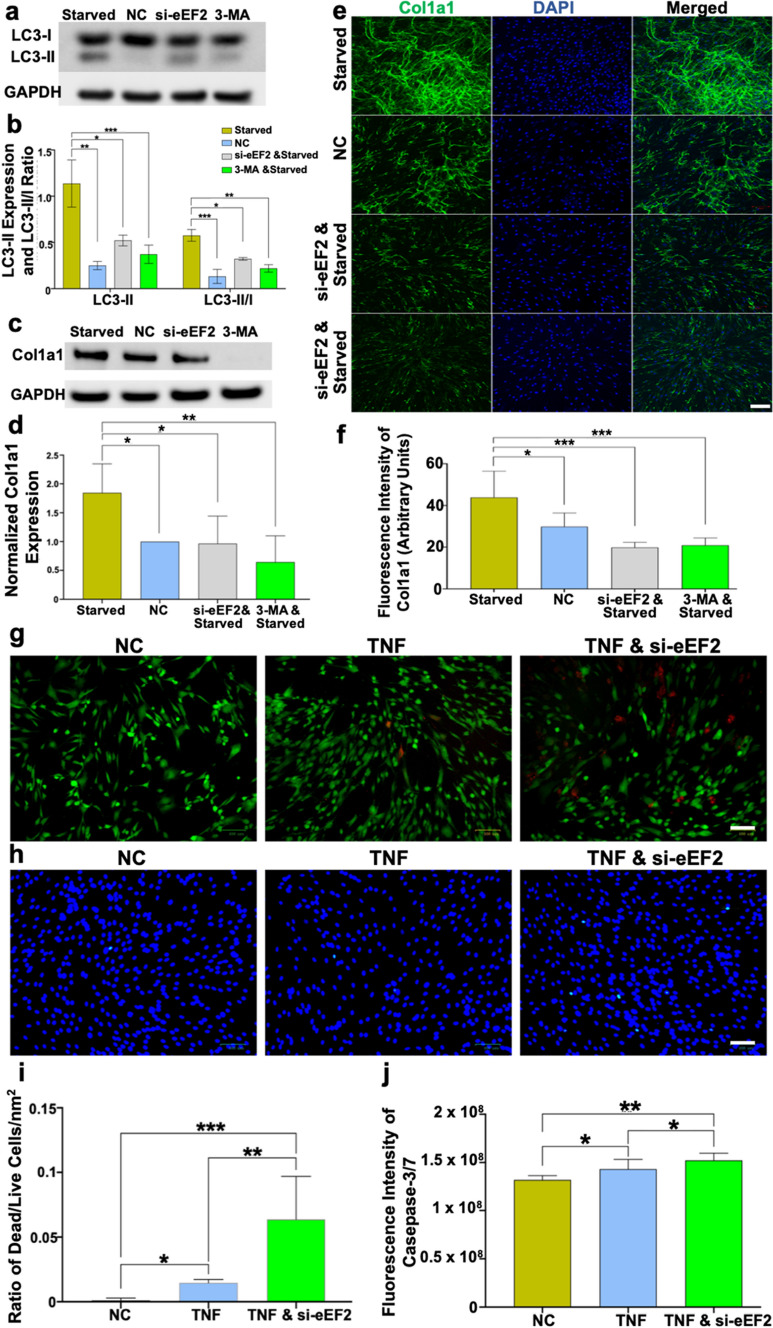
eEF2 enhances healing processes in TNF-induced inflammatory fibroblast models. **a**–**f** eEF2 affects Col1a1 expression through autophagy in human fibroblasts. **a**, **b** Representative Western blot images and semi-quantitative analysis of LC3-II and LC3-II/LC3-I ratio, **c**–**f** representative Western blot (**c**) and confocal images (**e**) along with semi-quantitative analysis of Col1a1 (**d**, **f**) in cells treated with normal medium, starved medium for autophagy, si-eEF2 and 3-MA incubation based on autophagy, TNF was used afterward to create an inflammatory model; signal intensity (**a**–**d**) and fluorescent green intensity (**e**, **f**) were used for semi-quantitative analysis; **g**–**i** representative images captured by fluorescent microscope demonstrated the cell death and apoptosis when treated with normal condition, TNF and si-eEF2 and TNF: si-eEF2 increases the ratio of dead/live cells among primary fibroblast and fibroblast cell line; si-eEF2 positively associate with cell apoptosis among human fibroblasts **h**, **j**; the ratio of dead/live cells was reported by percentage and the apoptotic level of cells was presented by fluorescent green intensity. Data reported as mean $\pm$ SD, *p < 0.05, **p < 0.01, ***p < 0.001, scale bars = 100 µm, three replicates were used for quantitative analysis

Autophagy was induced in the human fibroblast cell line and in primary fibroblasts, leading to an increase in microtubule-associated proteins light chain 3-II (LC3-II) and the ratio of LC3-II/I. The expression of LC3-II and the ratio of LC3-II/I in the fibroblast cell line TERT166 and in primary fibroblasts were significantly reduced when eEF2 was knocked down with si-eEF2, suggesting a positive effect of eEF2 on autophagy (Fig. 4a, b). Further exploration of this relationship demonstrated that autophagy led to increases in Col1a1 expression and that this upregulation was significantly reduced by knocking down eEF2 (Fig. 4c–f). Interestingly, the up- or downregulation of autophagy and subsequent Col1a1 production resulting from si-eEF2 was approximately equal to the effect induced by an autophagy inhibitor (3-MA) (Fig. 4e, f), demonstrating an essential impact of eEF2 on autophagy. Additionally, we observed that primary fibroblasts and the fibroblast cell line yielded very similar results, findings which strengthened the finding regarding the effect of eEF2 on autophagy during DCT repair (Supplementary Fig. 5).

These experimental findings confirmed and expanded the results of the bioinformatic analysis regarding the relationship between eEF2 and autophagy. To our knowledge, this is the first exploration of mechanistic function of eEF2 during regenerative process, highlighting that eEF2 improves Col1a1 production potentially by upregulating autophagy during inflammation.

### eEF2 enhances dense connective tissue repair during inflammation by reducing cell death and apoptosis

After tissue injury, the inflammatory stage of healing is comprised of multiple biological processes that are crucial toward tissue repair [31–33]. Among these processes, we identified that eEF2 not only improved autophagy but also reduced apoptotic processes during tissue repair (Supplementary Fig. 3). To confirm the preliminary findings from the bioinformatic and statistical analysis, the ratio of dead/live cells was assessed in two si-eEF2 inflammatory fibroblast models.

The experimental data demonstrated that induction of inflammation led to increase in the ratio of dead/live cells for both primary fibroblasts and the fibroblast cell line (Fig. 4g, i). Further, TNF-induced an upregulation of dead/live ratio that was even higher than when eEF2 was silenced (Fig. 4g). In addition, the increased ratio of dead/live fibroblasts lead to higher rates of apoptotic cells as observed by caspase-3/7 activity (Fig. 4h, j). All the results from primary fibroblasts and the cell line are shown in Supplementary Fig. 5. Hence, this stepwise assessment corroborated that eEF2 decreases cell death and reduces the apoptotic process, highlighting eEF2 as a multi-functional biomarker of tissue repair during the inflammatory stage. Moreover, identical findings from the primary fibroblasts and the fibroblast cell line strengthen the concept that eEF2 plays a vital role by reducing cell death/apoptosis during the inflammatory phase of DCT repair.

### eEF2 improves dense connective tissue repair by enhancing cell proliferation

DCTs repair is a complex process in which the inflammatory phase of healing is successively replaced by proliferative healing processes and matrix deposition. Our recent finding based on the proliferative stage of healing [12] also detected an increased eEF2 expression in the healing tendons at 2 weeks post repair surgery. Interestingly, analysis of the MS-based quantitative proteomic data from the early proliferating healing phase; 2-weeks post-surgery, detected elevated levels of eEF2 in the healing compared to intact Achilles tendons (Supplementary Fig. 6). To further understand the role of eEF2 on proliferative healing processes, the effects of si-eEF2 on proliferating fibroblasts in unchallenged cell line as well in primary fibroblasts were studied.

**Fig. 5:**
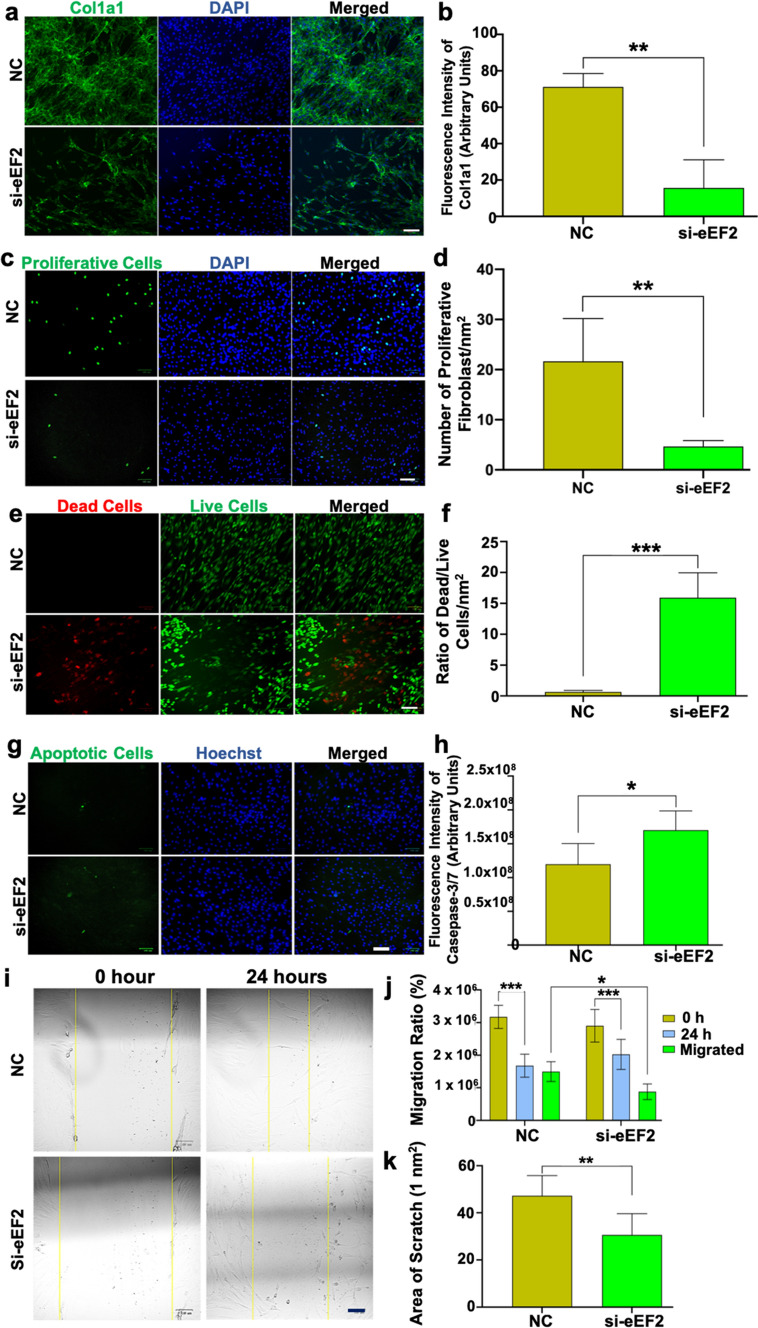
eEF2 enhances fibroblast proliferative processes. **a**, **b** Representative confocal images and semi-quantitative analysis of Col1a1 in primary fibroblasts and fibroblast cell line, with and without si-eEF2; **c**, **d** representative immunofluorescence images and number of proliferating human fibroblasts, with and without si-eEF2; **e**, **f** representative immunofluorescence images and ratio of dead/live fibroblasts, with and without si-eEF2; **g**, **h** representative immunofluorescence images and number of apoptotic cells from primary fibroblasts and fibroblast cell line, with and without si-eEF2; **i**–**k** representative images and quantitative analysis of cell migration rate assessed at 0 and 24 h in primary fibroblast and cell line, with and without si-eEF2. Data reported as mean $\pm$ SD, *p < 0.05, **p < 0.01, ***p < 0.001, scale bars = 100 µm, *n* = 3 replicates

Our first analysis showed a decline in Col1a1 production by knocking down eEF2 (Fig. 5a, b), suggesting an association of eEF2 to this repair-related matrix protein. Quantitative image analysis demonstrated an eEF2-induced cell proliferation among cells treated with si-eEF2 (Fig. 5c, d). These observations confirmed and strengthened the preliminary finding, and also extended on the potential role of eEF2 from inflammatory to the early proliferative stages of tendon tissue repair.

### eEF2 improves dense connective tissue repair by reducing cell death and apoptosis during proliferation

Cell death is an essential yet opposing biological process in relation with proliferation [34, 35], while apoptosis is the outcome of cell death. However, the knowledge base for the potentially synchronized regulation of cell apoptotic- and proliferative processes during tissue repair is limited. For the next step of the analysis, the impact of eEF2 on the coordinated cell proliferation and apoptosis leading to connective tissue repair was explored using unchallenged fibroblast models.

The experimental observations indicate an opposite effect of eEF2 on cell death when compared with cell proliferation as demonstrated by increases in the ratio of dead/live cells when cells were treated with si-eEF2 (Fig. 5e, f). The pathway to cell death was corroborated by demonstrating an increased apoptotic ratio of cells following treatment with si-eEF2 (Fig. 5g, h).

Here, these findings for the first-time report that eEF2 induces fibroblast proliferation, and at the same time reduces cell death and apoptosis to improve connective tissue repair and outcomes during an early phase of healing. These observations extended the role of eEF2 at cellular level, but also highlighted its mechanistic function during the early proliferative phase of tissue repair.

### eEF2 improves dense connective tissue repair by enhancing cell migration during proliferation

**Fig. 6:**
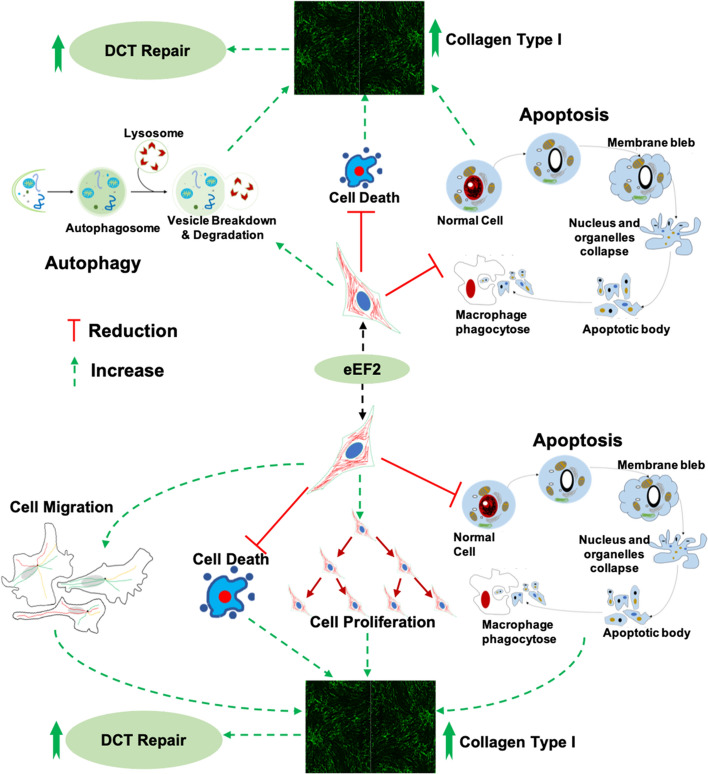
Potential mechanisms and eEF2 mode of action during inflammation and proliferation phases of dense connective tissue repair

During wound healing, fibroblast migration to the site of injury is a crucial step to initiate the proliferative healing process [36]. Our bioinformatic analysis also observed that eEF2 expression was positively associated with cell migration molecules (Supplementary Fig. 3). Thus, we assessed whether eEF2 enhances cell migration during the proliferation phase of tissue healing. For these experiments, in-vitro wound models were used in which a scratch was created in monolayer cultured fibroblasts using both the primary cells and the cell line, treated with or without si-eEF2. The findings confirmed the earlier bioinformatic analysis by demonstration of a reduced cell migration area and ratio among cells transfected with si-eEF2 (Fig. 5i–k). Quantitative analysis of the results demonstrated a significantly (*p* = 0.01) slower (30%) migration rate in eEF2 knock down cells in comparison with a 47% rate in normal cells when primary cells were used. When the cell line was used in studies, the migration rate was 33% in the si-eEF2 treated cells versus a 55% rate in the control cells (Supplementary Fig. 6). The consistent findings between primary fibroblasts and a fibroblast cell line further support a mechanistic role for eEF2 in cell migration during connective tissue repair, all the results based on outcomes resulting from the use of this dual cell line confirmatory approach are presented in the supplementary materials (Supplementary Fig. 6).  The detailed mechanisms of this study about how eEF2 affects DCT repair is presented in the following Fig. 6.

## Discussion

DCTs are essential to support, protect, and give structure to other tissues and organs in the body. After injury, a reparative process is initiated in dense connective tissues which is highly dependent on protein synthesis. However, biomarkers predictive of good patient outcomes after connective tissue (CT) repair are lacking, particularly those detected during the early phases of the healing process. In this study, the proteomic composition of DCT repair was quantitatively characterized in patients with acute Achilles tendon rupture that were surgically repaired as an example of a clinical relevant DCT. We successfully generated a comprehensive proteomic file of good versus poor outcome patients. This cohort constitutes a novel collection of human proteomic data related to validated clinical outcomes. These findings obtained from injured Achilles tendon may serve as a representative of more general DCT healing and thus, could greatly improve our understanding of the healing processes involved in DCT repair. We assume that future mining of this proteomic file will expand our understanding of dense connective tissue repair to identify additional potential pathways and potential targets lead to enhancement of healing.

The enrichment analysis of proteomic data identified differentially expressed proteins, which mainly were involved in collagen binding, ECM regulation and organization, and wound healing via cell migration, proliferation and apoptosis. Further exploration of the proteomic file based on a validated clinical database identified a novel biomarker, eEF2 with strong predictability of good healing outcome. By combining bioinformatic analysis along with experimental investigations, the role of eEF2 during tissue repair was explored and verified. Mechanistic exploration of eEF2 identified its potential in vivo role in enhancing autophagy, cell migration and proliferation, as well as reducing cell death and apoptotic processes, all of which contribute to an improved wound healing process.

DCT repair consists of dynamic and overlapping phases, including inflammatory, proliferative and regenerative phases [37]. To understand the mechanisms of eEF2 in DCT repair we, in a stepwise manner, investigated how this biomarker regulates tissue healing from the inflammatory to the early proliferative healing processes. The finding that eEF2 increases Col1a1 production by positively regulating fibroblast autophagy during inflammation is supported in the literature among different cell types showing an association between autophagy and Col1a1 production [29, 38–40]. eEF2 is an elongation factor, which promotes the GTP-dependent translocation of the ribosome, and thus presumably also promotes Col1a1 synthesis in CT repair [24, 41]. Col1a1, produced by fibroblasts, encodes the major component of type I collagen, the most abundant collagen in the ECM of healthy connective tissues [42–44]. Moreover, higher expression of type I collagen leads to improved CT repair [15]. Thus, our combined bioinformatics analysis and mechanistic approaches using inflammatory fibroblast models created with both primary human cells and a human cell line, confirmed the hypothesis that eEF2 enhances dense connective tissue repair during the inflammatory phase of healing by increasing autophagy and thereby promoting the production of Col1a1.

The bioinformatic analysis also disclosed a negative relationship between the proteomic profile and apoptosis, which is supported by earlier studies showing that autophagy and apoptosis inhibit each other by protein cleavage or degradation in an enzyme dependent manner [45]. In addition, pre-clinical studies have indicated that the inhibition of eEF2 in mice and rat models were found to reduce autophagy, but at the same time activate cell death and apoptosis, as well as decrease protein production in skeletal tissue [46, 47]. The current findings that eEF2 reduces cell apoptosis as indicated by the downregulation of the ratio of dead/live cells and apoptotic cells not only extend the role of eEF2 from animal to human species, but also provide mechanistic insight for its role in enhancing DCT repair during the inflammatory phase of healing.

Cell proliferation is the subsequent phase in wound healing [48–50], in which eEF2 has been reported to act as a promoter [51], presumably by improving the cell proliferative process through the PI3K pathway [52]. However, whether eEF2 plays a key role during the proliferation phase of tissue repair remains unclear. The identification of increased eEF2 levels also at 2-weeks post Achilles tendon surgery [12], suggests a role in the early proliferative healing phase as defined in earlier studies of Achilles tendon healing [16, 53]. Fibroblasts play a vital role during proliferation [54–57]. Here, we used both unchallenged human primary fibroblasts and a fibroblast cell line, mimicking aspects of the proliferation phase of healing, to investigate the mechanistic role(s) of eEF2 in tissue repair. The combined bioinformatic and mechanistic analysis confirmed that eEF2 enhanced fibroblast proliferation and increased Col1a1 production. In addition, the positive association between eEF2 and Col1a1 extends our understanding of the role of this protein in tissue healing. The coordination between cell proliferative and apoptotic activity also plays a key role in tissue development and homeostasis in the human body [34]. The current findings that eEF2 increases cell proliferation and migration, and at the same time reduces cell death and apoptotic processes, suggest that eEF2 is not only a powerful biomarker, but also provides mechanistic insight of how eEF2 can regulate and enhance DCT repair during the proliferative phase. To the best of our knowledge, this is the first observation of eEF2 as a promoter of multiple phases of tissue repair.

## Conclusion

Taken together, the current findings present eEF2 as a marker prognostic of good quality human Achilles tendon repair, potentially generic to dense connective tissue repair. The bioinformatic and mechanistic analyses confirm that eEF2 promotes healing by regulating processes during the inflammatory as well proliferative phases of tissue repair. The findings presented may lead to the development of targeted treatments which could enhance the long-term healing outcomes for patients suffering with dense connective tissue injuries that currently yield a poor clinical outcome.

## Reporting summary

Further information on research design is available in the Nature Research Reporting Summary linked to this article.

### Supplementary Information

Below is the link to the electronic supplementary material.

Supplementary file1 (XLSX 1261 KB)

Supplementary file2 (DOCX 9003 KB)

Supplementary file3 (DOCX 3724 KB)

Supplementary file4 (DOCX 5081 KB)

Supplementary file5 (DOCX 6895 KB)

Supplementary file6 (DOCX 4085 KB)

## Data Availability

MS proteomic data files are deposited at the ProteomeXchange Consortium (http://proteomecentral.proteomexchange.org) through the PRIDE partner repository (https://www.ebi.ac.uk/pride/) under the dataset PXD029202 and PXD033163. The extracted protein LFQ intensity and GSEA data are provided as supplementary files. Clinical data was collected from the database at the section of Orthopedics, Department of Molecular Medicine and Surgery, Karolinska Institutet.
