# Supplementary figures and images for: eEF2 improves dense connective tissue repair and healing outcome by regulating cellular death, autophagy, apoptosis, proliferation and migration
Source: Cell Mol Life Sci. 2023 Apr 21;80(5):128. doi: 10.1007/s00018-023-04776-x (PMC10121543; doi:10.1007/s00018-023-04776-x)

Original Western blot image of Col1a1 in human tissue, n=18


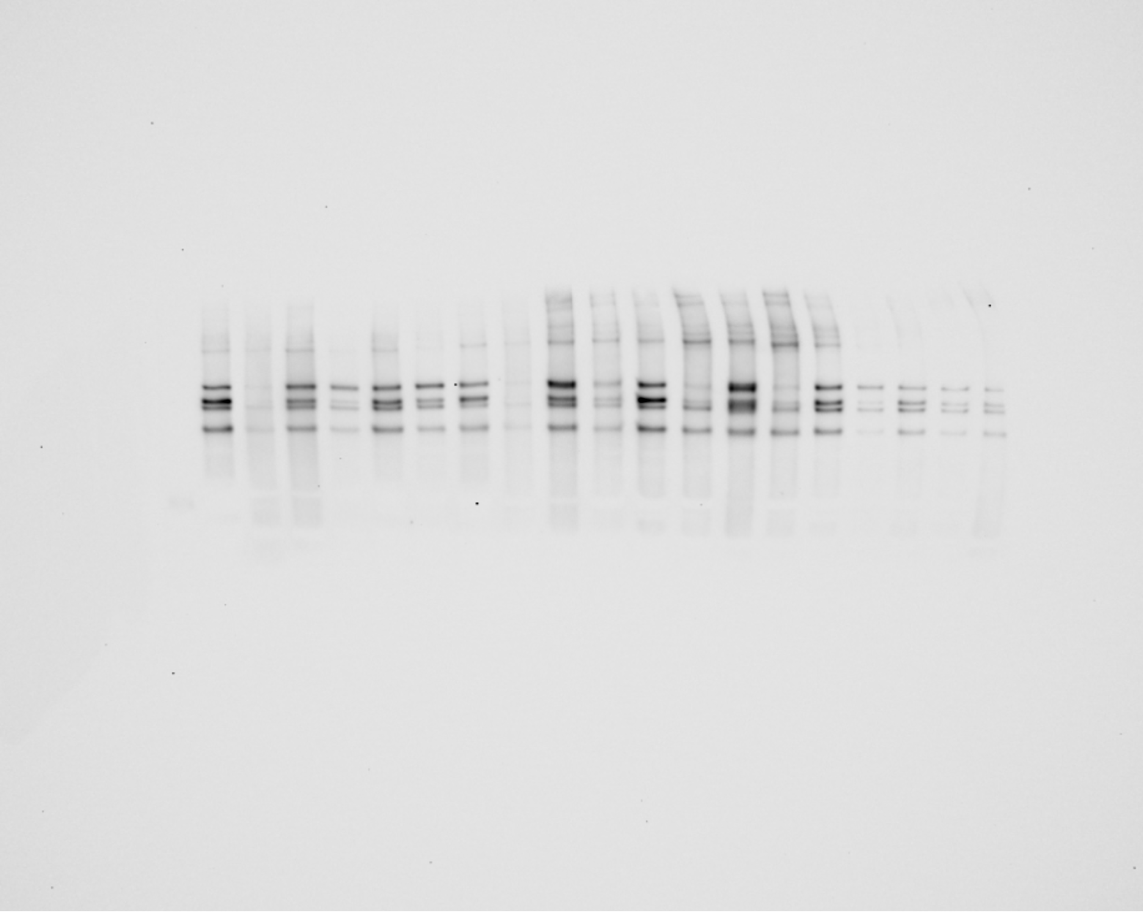

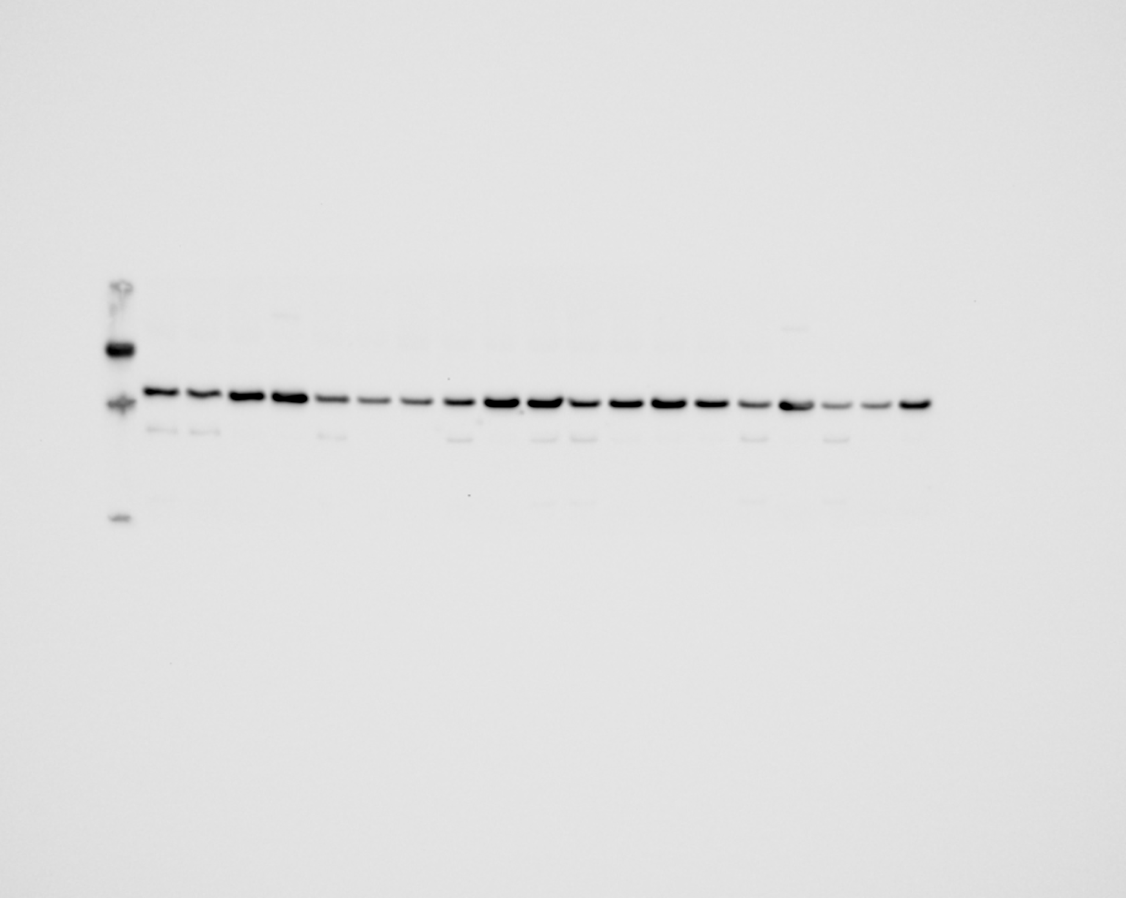


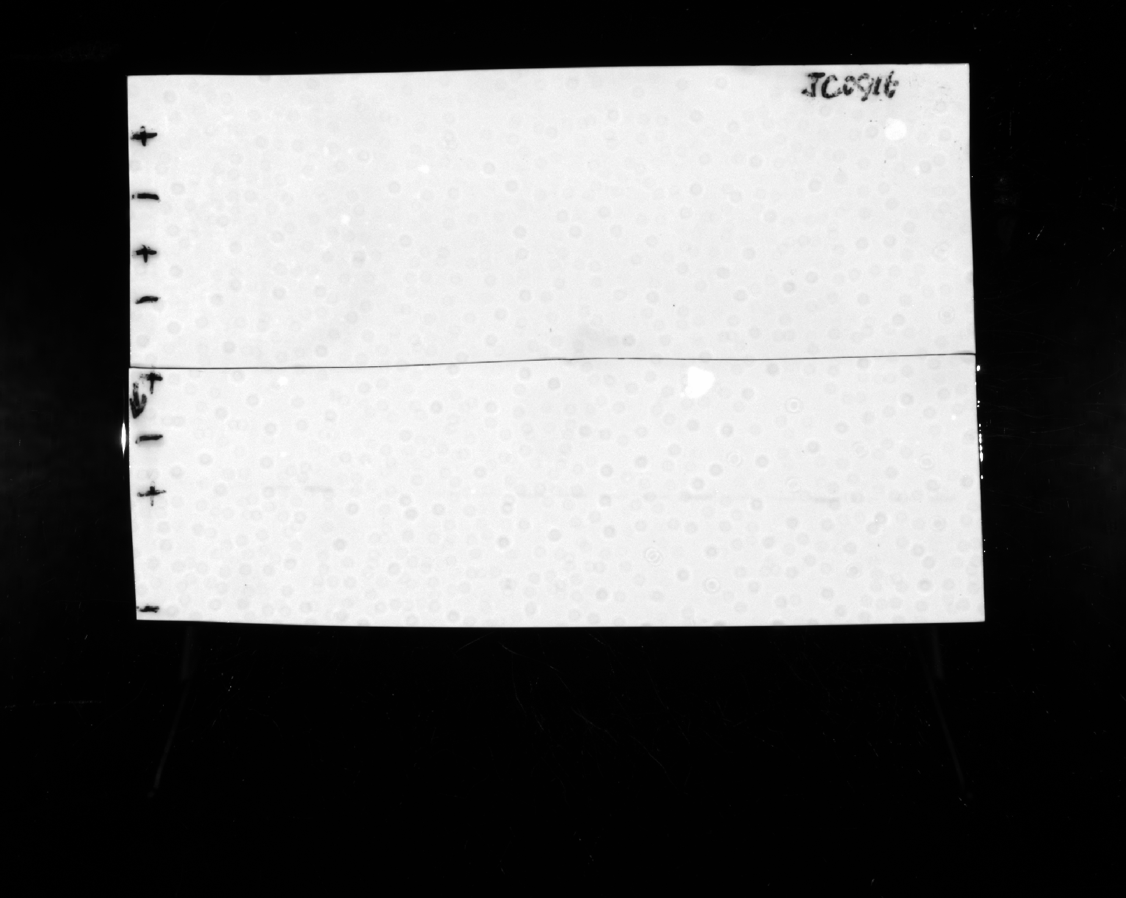

Supplement: Supplementary file 2 — Supplementary file2 (DOCX 9003 KB) [file 18_2023_4776_MOESM2_ESM.docx]

Original Western blot image of eef2 in human tissues, n=18


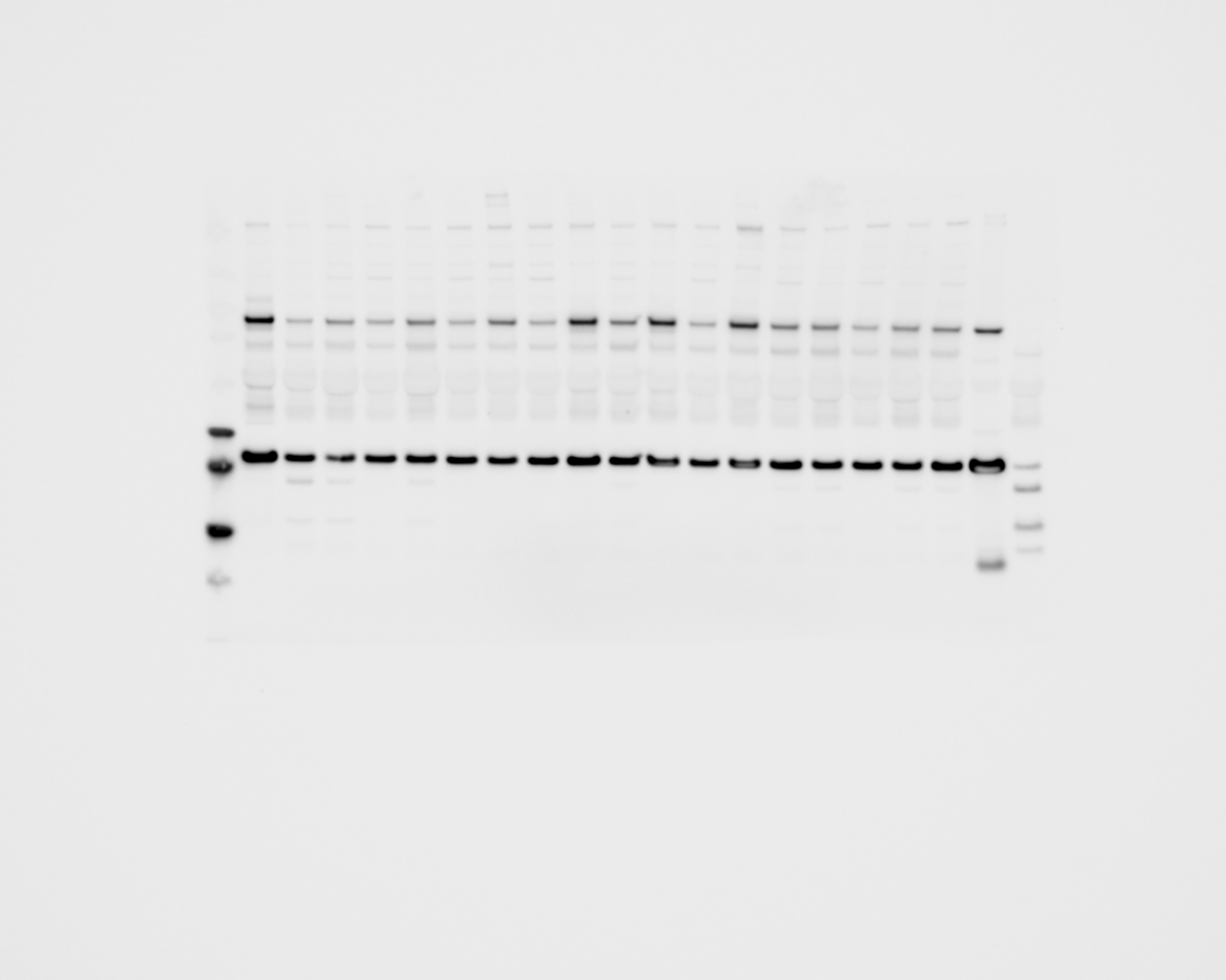

Supplement: Supplementary file 3 — Supplementary file3 (DOCX 3724 KB) [file 18_2023_4776_MOESM3_ESM.docx]

Original Western blot image of LC3-I and II in primary fibroblast and fibroblast cell line:


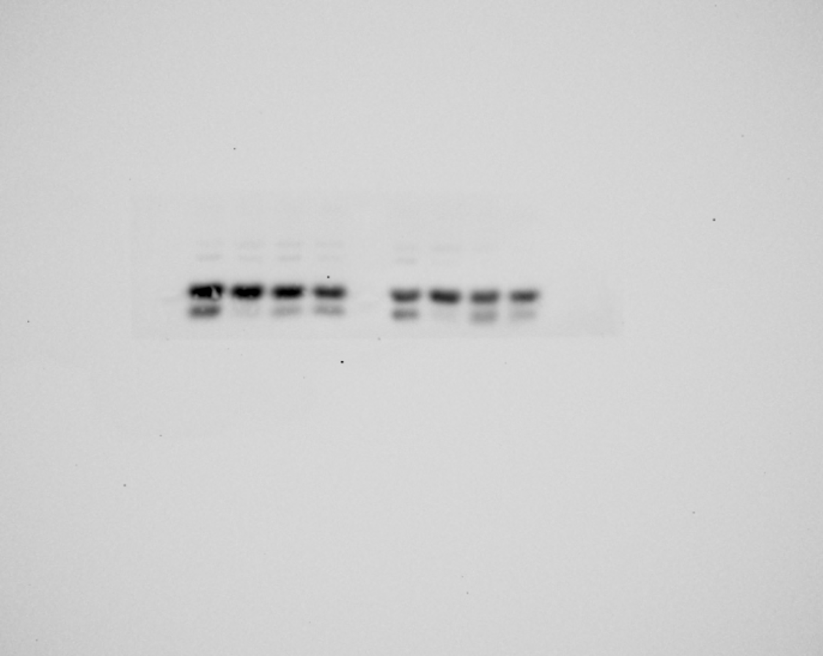


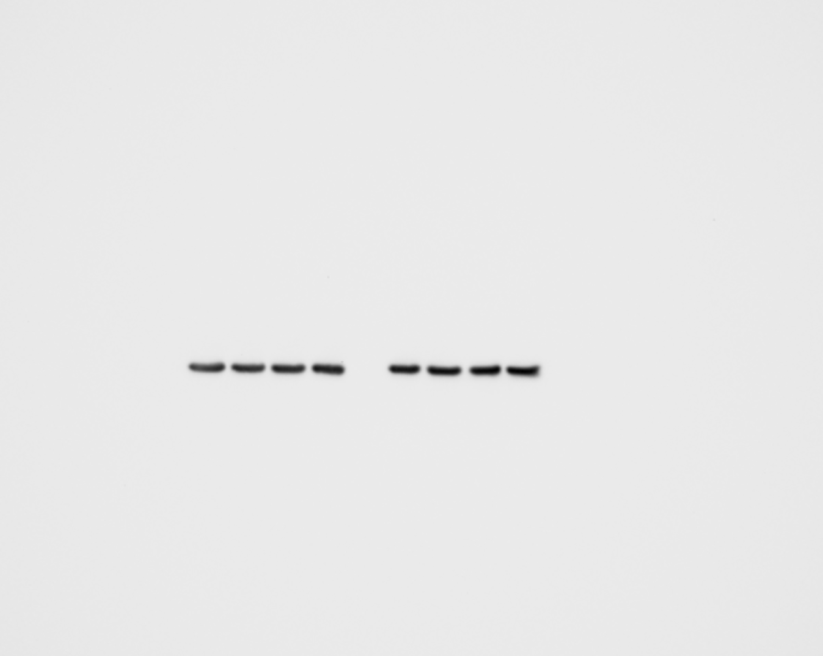


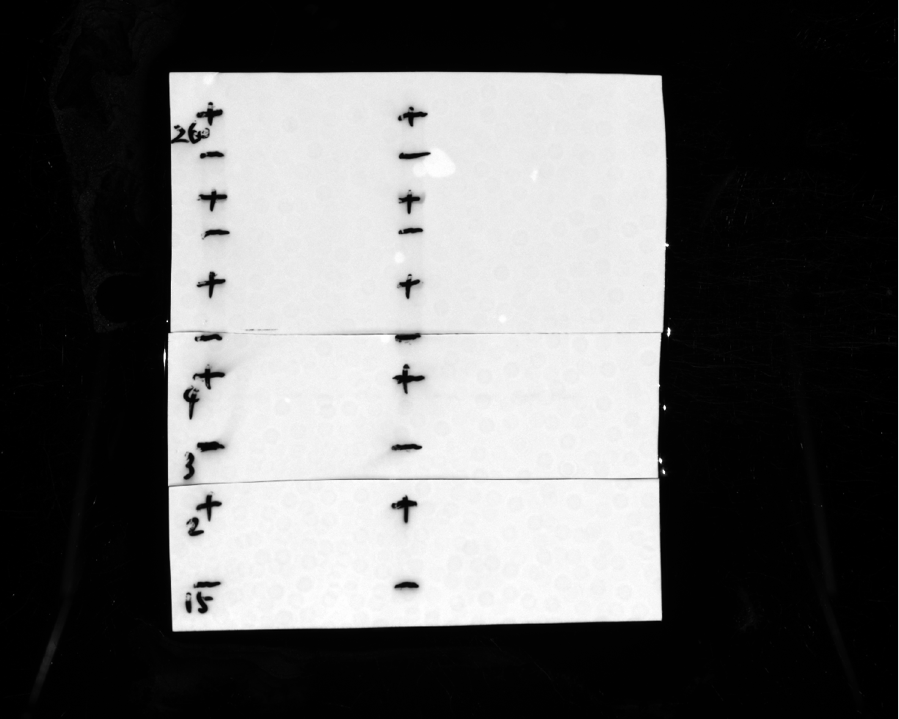

Supplement: Supplementary file 4 — Supplementary file4 (DOCX 5081 KB) [file 18_2023_4776_MOESM4_ESM.docx]

Original Western blot image of Col1a1 (220kDa) in primary fibroblast and fibroblast cell line:


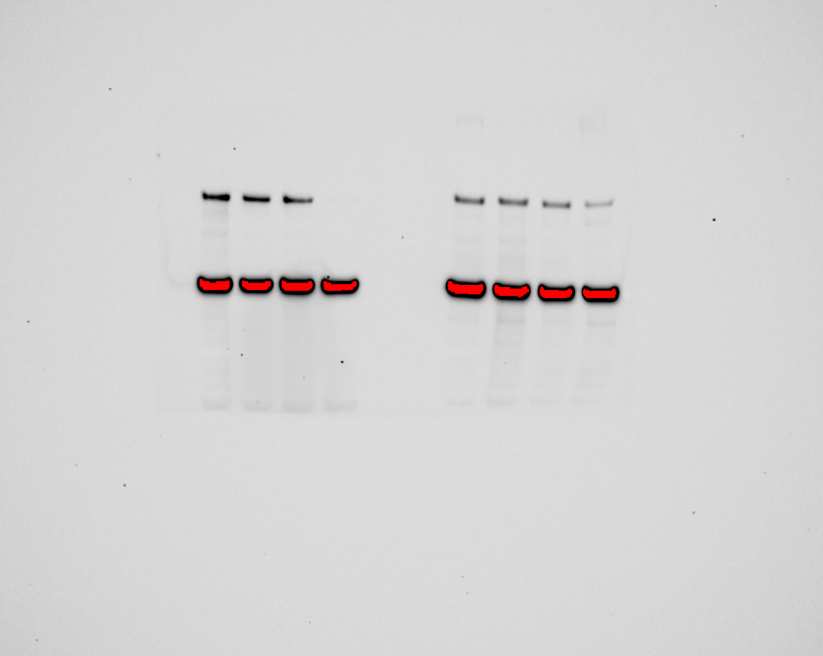


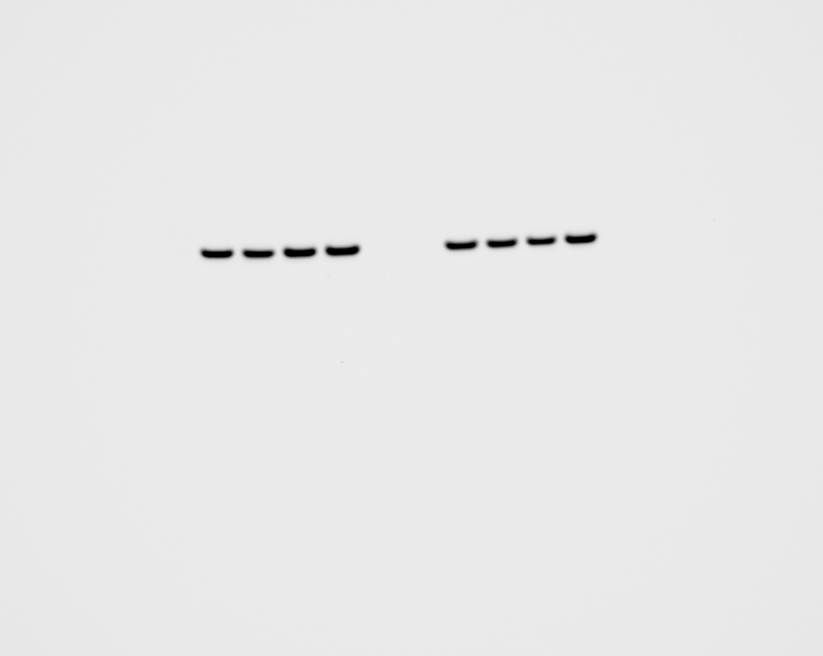


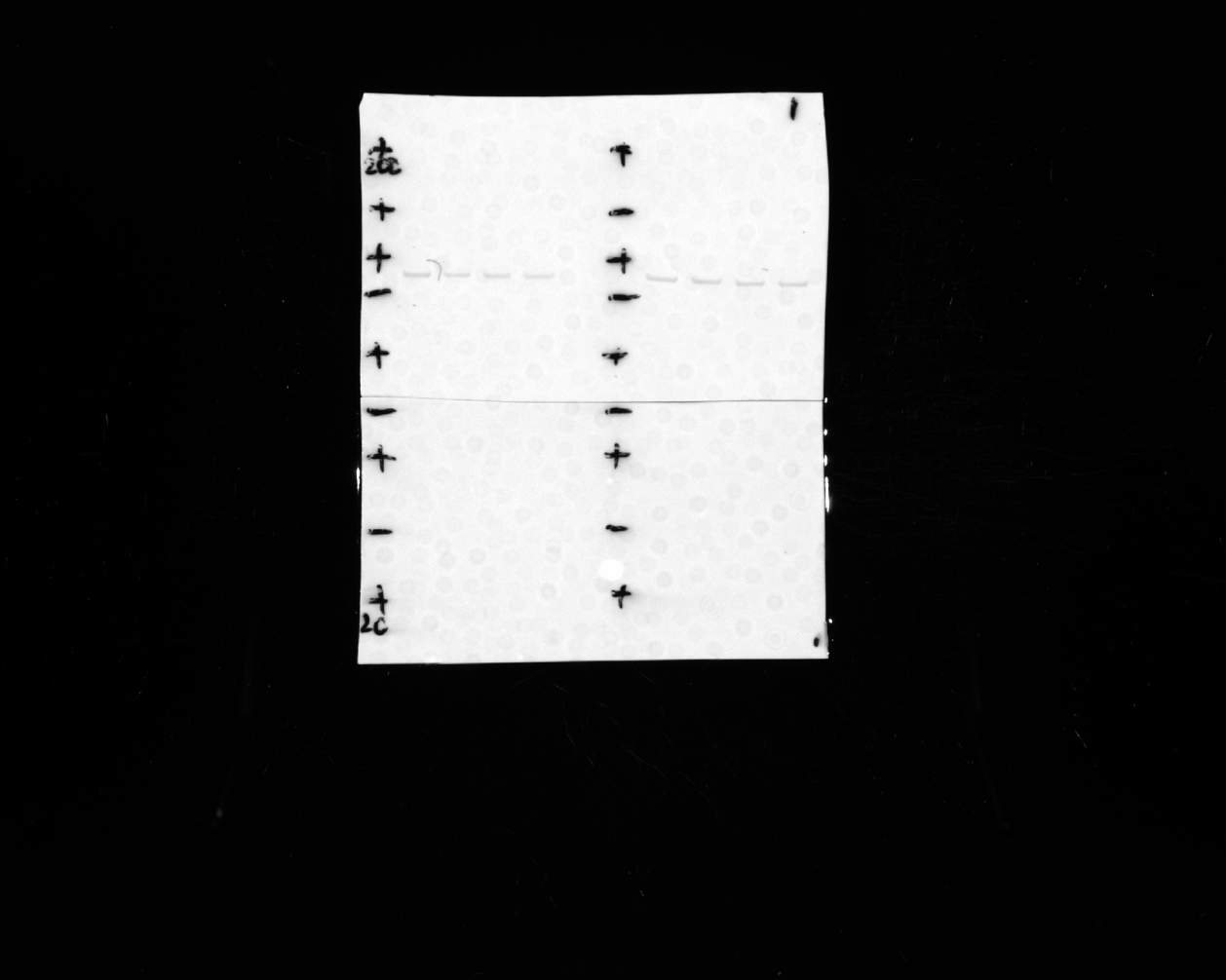

Supplement: Supplementary file 5 — Supplementary file5 (DOCX 6895 KB) [file 18_2023_4776_MOESM5_ESM.docx]
