# Supplementary material for: eEF2 improves dense connective tissue repair and healing outcome by regulating cellular death, autophagy, apoptosis, proliferation and migration
Source: Cell Mol Life Sci. 2023 Apr 21;80(5):128. doi: 10.1007/s00018-023-04776-x (PMC10121543; doi:10.1007/s00018-023-04776-x)
Supplement: Supplementary file 6 — Supplementary file6 (DOCX 4085 KB) [file 18_2023_4776_MOESM6_ESM.docx]

**Supplementary Figure 1(Fig S1).**

**
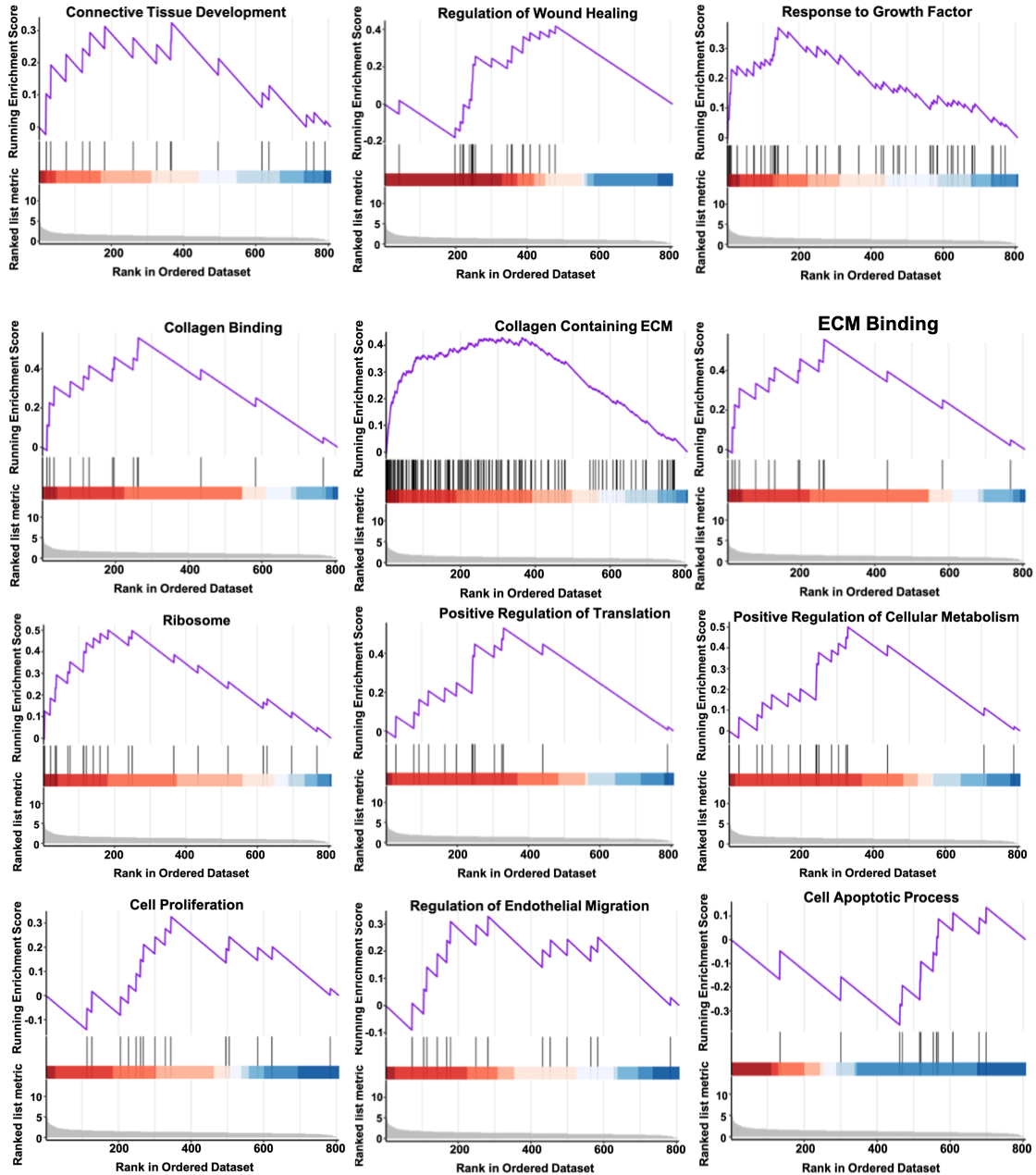
**

**Fig S1.** All the potential pathways which are involved with 51 (10 down- and 41 up-regulated) differentially expressed proteins based on GSEA.

**Supplementary Figure 2 (Fig S2).**


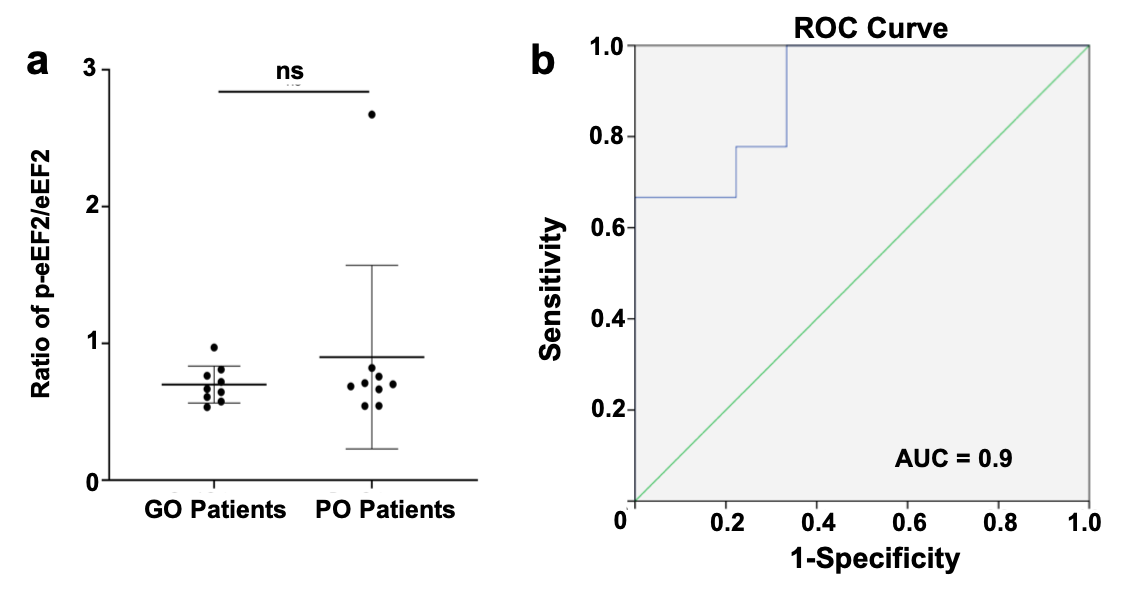


**Fig S2.** a) Semi-quantitative analysis based on western blotting result among 18 patients, the ratio of phosphorylated-eEF2 (p-eEF2)/total eEF2 shows no statistical significance between good- and poor outcome patients, n = 18; b) AUC for eEF2 to report its predictive significance based on Western Blot result, n = 18.

**Supplementary Figure 3 (Fig S3).**


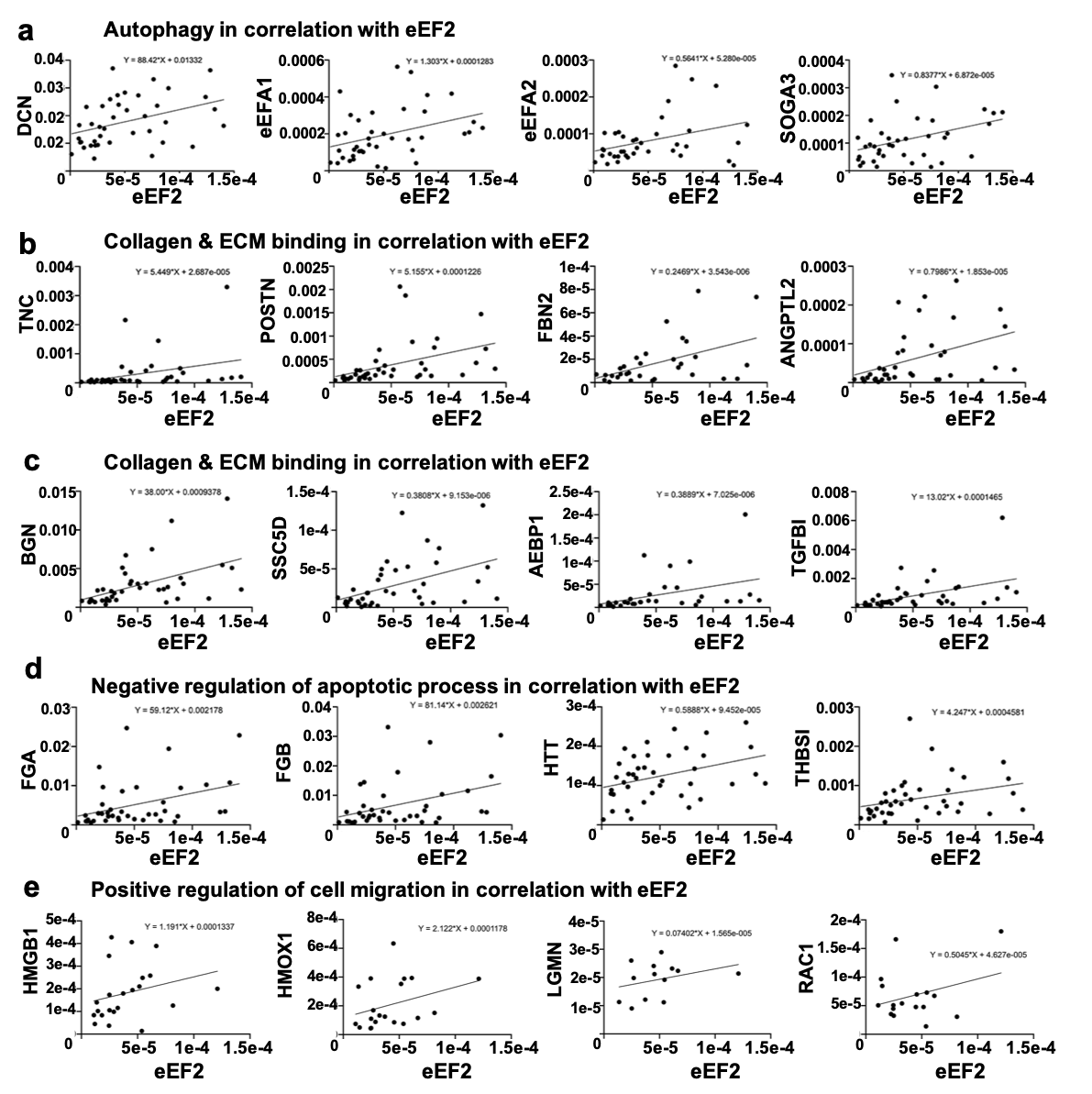


**Fig S3.** Bioinformatics of eEF2 in relation to inflammatory stage of healing.

a) A positive correlation between eEF2 and autophagy; b-c) There was a positive association between collagen & ECM binding and eEF2; d) eEF2 was positively associated with regulation of apoptosis; e) eEF2 was positively associated with cell migration. All the proteins which were enriched in autophagy, collagen & ECM binding, negative regulation of apoptotic process and positive regulation of cell migration were collected and used a regression analysis with eEF2.

**Supplementary Figure 4 (Fig S4).**


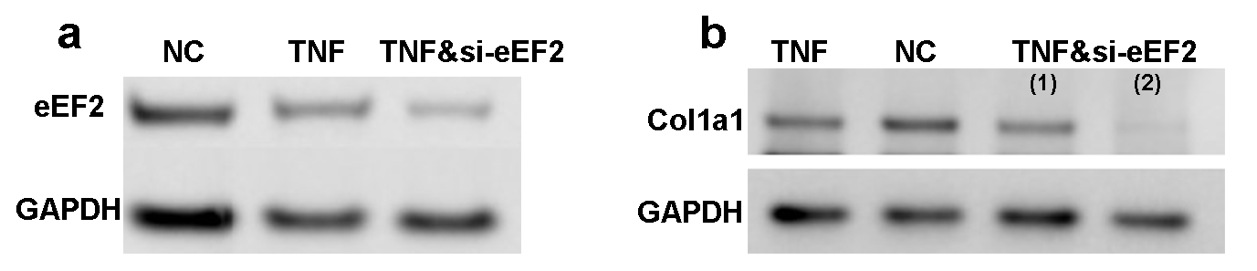


**Fig S4.** eEF2 knock down among the inflammatory fibroblast model.

a) Representative images of western blotting from proteins lysates generated from fibroblasts treated with normal medium, TNF stimulation and si-eEF2 incubation after TNF stimulation. Cells treated with TNF showed a 22% decrease in eEF2 expression while si-eEF2 incubated cells demonstrated a 67% decrease in eEF2 expression.

b) Representative images of western blotting from proteins lysates generated from fibroblasts treated with normal medium, TNF stimulation and si-eEF2 incubation after TNF. Cells incubated with TNF showed a 37% decrease of Col1a1 production when compared with normal control. Cells showed a 50% (48 hours) decrease and 87% (72 hours) decrease in Col1a1 production when treated with si-eEF2 after incubating with TNF.

**Supplementary Figure 5 (Fig S5).**


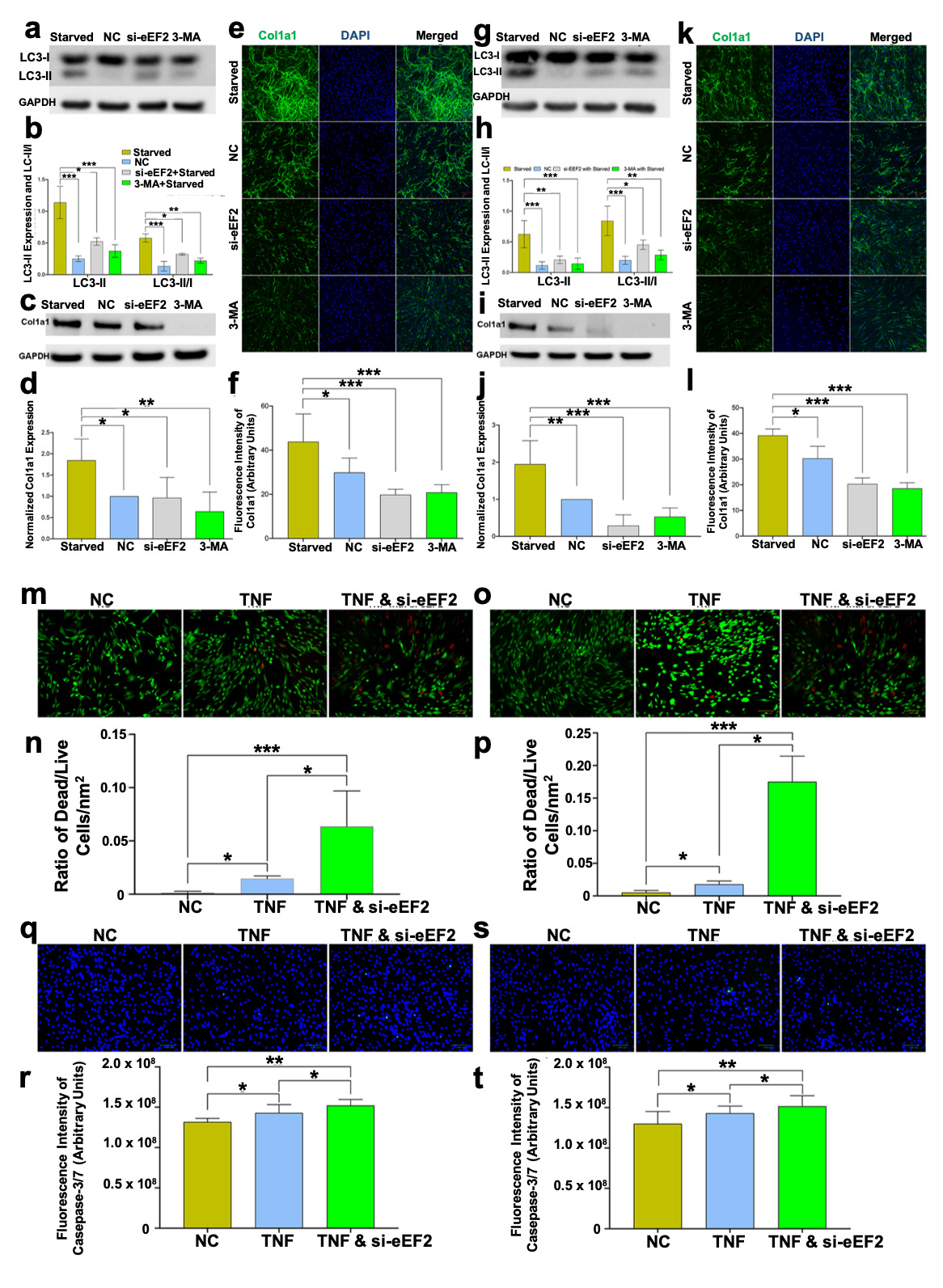


**Fig S5.** eEF2 enhances healing processes in TNF-induced inflammatory fibroblast models.

a-f) eEF2 affects Col1a1 expression through autophagy in primary fibroblasts. a, b) Representative Western blot images and semi-quantitative analysis of LC3-II and LC3-II/LC3-I ratio, c-f) Representative Western blot (c) and confocal images (e) along with semi-quantitative analysis of Col1a1(d, f) in cells treated with normal medium, starved medium for autophagy, si-eEF2 and 3-MA incubation based on autophagy; g-l) eEF2 affects Col1a1 synthesis by autophagy in fibroblast cell line. g, h) Representative Western blot images and semi-quantitative analysis of LC3-II and LC3-II/LC3-I ratio. Representative Western blot (i) and confocal images (k) along with semi-quantitative analysis of Col1a1 (l, j) synthesis in cells treated with normal medium, starved stimulation for autophagy, si-eEF2 and 3-MA incubation based on autophagy; Semi-quantitative analysis demonstrated eEF2 enhanced autophagy and then increased Col1a1 synthesis during inflammation. Signal intensity (a-d, g-j) and fluorescent green intensity (e-f, k-l) were used for semi-quantitative analysis; m-t) Representative images captured by fluorescent microscope demonstrated the cell death and apoptosis when treated with normal condition, TNF and si-eEF2: si-eEF2 increases the ratio of dead/live cells among primary fibroblast (m, n) and fibroblast cell line (o, p); si-eEF2 positively associate with cell apoptosis among primary fibroblasts (q, r) and fibroblast cell line (s, t); The ratio of dead/live cells was reported by percentage and the apoptotic level of cells was presented by fluorescent green intensity. Data reported as mean $\pm$ SD, * *p* < 0.05, ** *p* < 0.01, *** *p* < 0.001, scale bars = 100 µm, 3 replicates were used for quantitative analysis.

**Supplementary Figure 6 (Fig S6).**


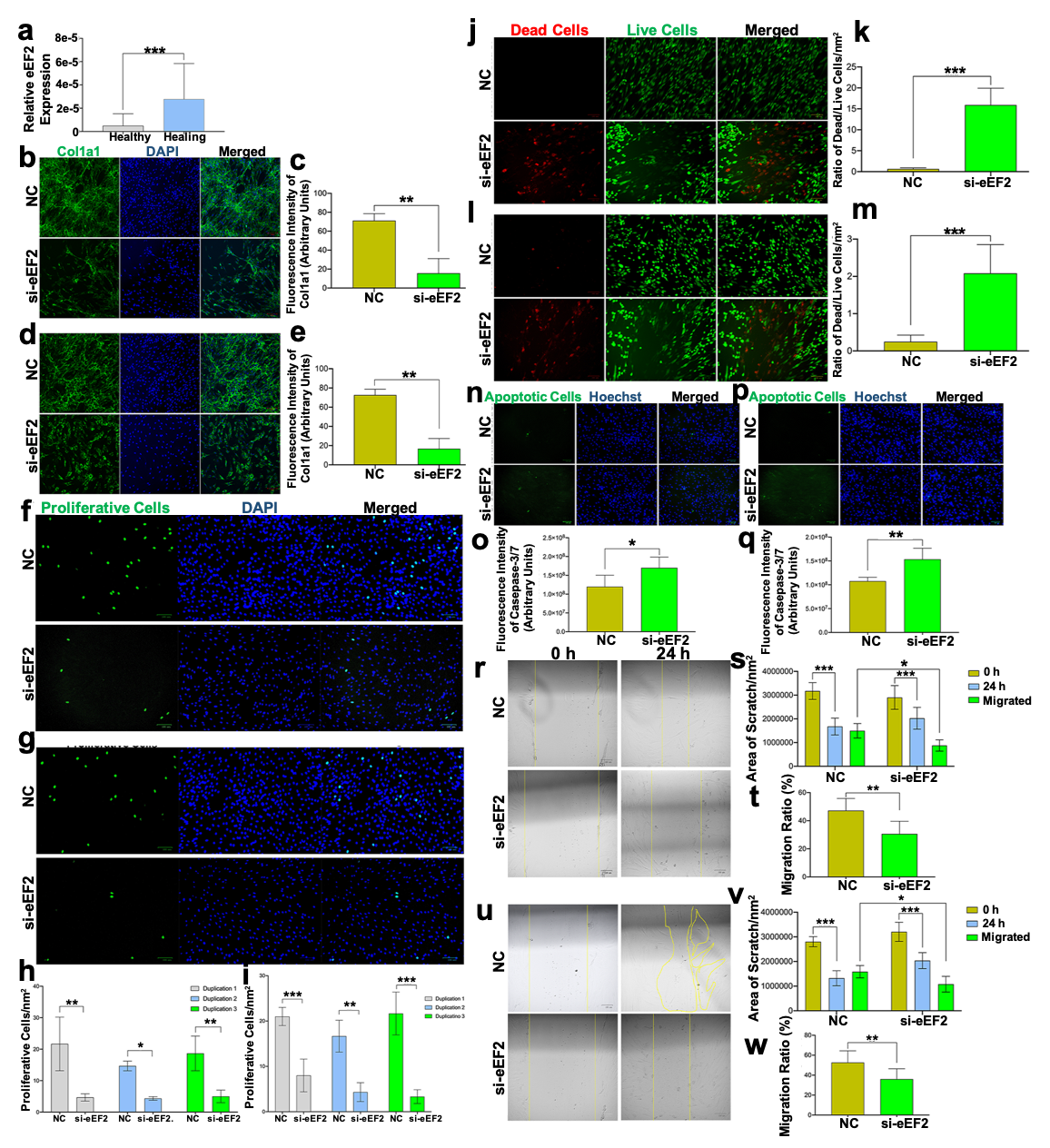


**Fig S6.** eEF2 enhances fibroblast proliferative processes.

a) eEF2 expression from micro-dialysate MS data, micro-dialysate collected from the intact and healing Achilles tendons, 2 weeks post-surgery, *** p < 0.001, n = 28 in each group; b-e) Representative confocal images and semi-quantitative analysis of Col1a1 in b,c) primary fibroblasts and, d,e) fibroblast cell line, with and without si-eEF2, data reported as mean $\pm$ SD, ** p < 0.01, scale bars = 100 µm, n = 3 replicates; f-i) Representative immunofluorescence images and number of proliferating f,h) primary fibroblasts and g,i) fibroblast cell line, with and without si-eEF2. Data reported as mean $\pm$ SD, * p < 0.05, ** p < 0.01, *** p < 0.001, scale bars = 100 µm, n =3 replicates; j-m) Representative immunofluorescence images and ratio of dead/live cells from, j,k) primary fibroblasts, and l,m) fibroblast cell line, with and without si-eEF2. Data reported as mean $\pm$ SD, *** p < 0.001, scale bars = 100 µm, n = 3 replicates; n-q) Representative immunofluorescence images and number of apoptotic cells from n,o) primary fibroblasts, and p,q) fibroblast cell line, with and without si-eEF2. Data reported as mean $\pm$ SD, * p < 0.05, ** p < 0.01, scale bars = 100 µm, n =3 replicates; r-w) Representative images and quantitative analysis of cell migration rate assessed at 0 and 24 hours in r-t) primary fibroblast, and u-w) fibroblast cell line, with and without si-eEF2. Data reported as mean $\pm$ SD, * p < 0.05, ** p < 0.01, *** p < 0.001, scale bars = 100 µm, n =3 replicates.
